# It’s not all DAT: harnessing the potential of organic cation transporter 3 inhibition to selectively attenuate amphetamine reinforcement and dopamine release

**DOI:** 10.1038/s41386-026-02390-6

**Published:** 2026-03-26

**Authors:** Lauren E. Honan, Briana Mason, W. Anthony Owens, Sangbin Shin, Yeon Ha Ju, Yonggong Shi, Rebecca E. Horton, Lief E. Fenno, Gregory T. Collins, Lynette C. Daws

**Affiliations:** 1 Department of Cellular & Integrative Physiology, University of Texas Health Science Center at San Antonio, San Antonio, TX, USA; 2 Department of Pharmacology, University of Texas Health Science Center at San Antonio, San Antonio, TX, USA; 3 Department of Neuroscience, University of Texas at Austin, Austin, TX, USA; 4 Department of Psychiatry and Behavioral Sciences, Dell Medical School at UT Austin, Austin, TX, USA; 5 South Texas Veterans Health Care System, San Antonio, TX, USA; 6 These authors contributed equally: Lauren E. Honan, Briana Mason

## Abstract

Afflicting over 50 million people worldwide and demonstrating growing global trends of abuse, amphetamine-type stimulant abuse poses a significant public health burden. No effective pharmacological treatments exist for amphetamine-type stimulant use disorders, underscoring a critical need to identify novel, effective therapeutic targets. Amphetamine exerts its actions in part by targeting high-affinity, low-capacity monoamine transporters, particularly the dopamine transporter (DAT). However, therapeutic interventions targeting DAT have been so far unsuccessful. Emerging evidence supports a role for the low-affinity, high-capacity organic cation transporter 3 (OCT3) in the actions of amphetamine. Here we use in vivo electrochemical and behavioral approaches, as well as constitutive and temporally-inducible OCT3 knockout mice, to establish OCT3 as a critical mediator of neurochemical and behavioral actions of amphetamine. We demonstrate that OCT3 substantially contributes to amphetamine-evoked dopamine efflux in dorsal striatum and is key to reinforcing effects of amphetamine in intravenous self-administration assays. Our novel findings provide convergent evidence to suggest that OCT3 plays a central role in mediating abuse-related effects of amphetamine, establishing OCT3 as a potential novel target for the development of therapeutics to treat amphetamine-type stimulant use disorders.

## INTRODUCTION

Abuse of amphetamine and derivative compounds is a major public health burden. With over 50 million users worldwide [1], overdose deaths associated with amphetamine-type stimulants are increasing and second in magnitude only to opioids in the U.S [2]. Importantly, there are no approved pharmacological treatments for amphetamine-type stimulant use disorder, underscoring a dire need to better understand mechanisms driving abuse- related properties of these drugs in order to identify novel targets and effective therapeutics.

Reinforcing effects of amphetamine are mediated largely via increased dopaminergic signaling in striatum. Amphetamine enters dopamine neurons through uptake at the dopamine transporter (DAT) as well as diffusion through the plasma membrane [3, 4]. As a DAT substrate, competitive inhibition of dopamine uptake is partially responsible for enhanced extracellular dopamine elicited by amphetamine in striatum. Once inside dopamine neurons, amphetamine acts to increase cytosolic pools of dopamine through interactions with dopamine synthesis, vesicular packaging, and metabolism [5–7]. Amphetamine then facilitates efflux of cytosolic dopamine by reversing the direction of dopamine transport at DAT [8, 9]. As such, DAT has been an attractive therapeutic target for treatment of amphetamine-type stimulant use disorders. Despite this, DAT inhibitors have so far been unsuccessful clinically to treat amphetamine-type stimulant use disorders [10], although methylphenidate and atypical DAT inhibitors have shown some potential [11]. Moreover, amphetamine-evoked dopamine release and conditioned place preference (CPP) persist in mice lacking DAT [12, 13], indicating that amphetamine elicits dopamine efflux and reward by one or more DAT-independent mechanisms.

Recently, a growing body of evidence implicates the low-affinity, high-capacity organic cation transporter 3 (OCT3) to play a role in regulating monoaminergic neurotransmission [3, 14–19]. OCT3 is a bidirectional monoamine transporter expressed in regions important to actions of amphetamine, including on striatal dopamine neurons [3, 20]. Further, gene polymorphisms of OCT3 have been associated with methamphetamine use disorder [21]. As such, it is plausible OCT3 may serve as a DAT-independent mechanism for amphetamine to increase dopamine and function as a reinforcer.

We have previously established a role for OCT3 in the neurochemical and behavioral actions of amphetamine [3, 19]. Briefly, inhibition of OCT3 with the non-selective OCT and plasma membrane monoamine transporter (PMAT) blocker decynium-22 (D22) attenuated amphetamine-induced hyperlocomotion and dopamine release in dorsal striatum [3]. These effects were lost in constitutive OCT3 knockout mice, suggesting OCT3 as the primary site of action to mediate effects of D22. Further, D22 abolished development of amphetamine CPP in wildtype mice, and OCT3 knockout mice did not develop amphetamine CPP [19]. These data suggest OCT3 may play a role in mediating the reinforcing effects of amphetamine, presumably via amphetamine-evoked dopamine efflux at OCT3 in addition to DAT, a premise we have provided evidence for through in vivo chronoamperometry experiments and subsequent ex vivo assays [3]. Using cultured superior cervical ganglion (SCG) preparations, which richly express OCT3 and the norepinephrine transporter (NET), we showed that amphetamine-induced efflux of tritiated MPP^+^ ([^3^H]MPP^+^) was only fully inhibited by a combination of the NET (DAT and serotonin transporter (SERT)) inhibitor cocaine and D22 [3]. This confirms a role of OCT3 in mediating amphetamine-stimulated substrate efflux. Taken together with our in vivo chronoamperometry findings, where inhibition of OCT3 in combination with DAT produced a more robust inhibition of amphetamine-evoked dopamine release than just inhibition of DAT alone [3], these data illustrate a significant role for OCT3 in the neurochemical and behavioral actions of amphetamine.

Here, we build on these findings to provide the first evaluation of amphetamine intravenous self-administration (IVSA) in constitutive OCT3 knockout mice. We found that OCT3 knockout, or pharmacological inhibition in wildtype mice, attenuated amphetamine IVSA, consistent with our previous CPP data [19]. Then, we generated mice with temporally inducible global OCT3 knockdown and replicated our findings illustrating the contribution of OCT3 to amphetamine-evoked dopamine release in dorsal striatum, providing further evidence that OCT3 is critical to the neurochemical actions of amphetamine. Importantly, we found that pharmacological inhibition of OCT3 may selectively attenuate amphetamine-evoked dopamine efflux, and subsequent reinforcing effects, without disrupting normal physiological function of the striatal dopamine system. Finally, we evaluated effects of D22 in nucleus accumbens (NAc) core, in which we found a lack of effect, contrary to our findings in dorsal striatum. These data suggest the contributions of OCT3 to the neurochemical actions of amphetamine may be striatal subregion-dependent. Together, our findings implicate OCT3 as a novel target for development of therapeutics to treat amphetamine-type stimulant use disorders.

## MATERIALS AND METHODS

Constitutive OCT3 wildtype (OCT3 WT) and knockout (OCT3 KO) mice (originating article: [22]) were obtained from an in-house colony at the University of Texas Health Science Center at San Antonio (UTHSCSA) and were used for all self-administration experiments. Tamoxifen-inducible OCT3 knockdown (OCT3 KD) mice were generated by crossing OCT3 floxed (OCT3^fl/fl^) mice, generated by the Mouse Genome Engineering and Transgenic Facility at UTHSCSA, with Gt(ROSA)26sortml(Cre/ERT2tyip)/J (R26^Cre^) mice (stock #008463, Jackson Laboratory, Bar Harbor, ME, USA, originating article: [23]) expressing Cre recombinase ubiquitously. These OCT3 knockdown mice were used for in vivo high-speed chronoamperometry experiments in dorsal striatum. Knockdown was induced with delivery of four 70 mg/kg injections (intraperitoneal, *i.p.*) of tamoxifen (Hello Bio Inc., Princeton, NJ, USA) dissolved in a 4:1 mixture of sunflower and castor oils. OCT3^fl/fl^ mice treated with tamoxifen were used as controls (OCT3 WT). Experiments were conducted 28 days post-tamoxifen. OCT3^fl/fl^ mice injected with AAV5-Ef1a-mCherry bilaterally in ventral tegmental area (VTA) were used for in vivo high-speed chronoamperometry experiments in NAc. All procedures were conducted in accordance with an approved Institutional Animal Care and Use Committee (IACUC) protocol and abided by current NIH guidelines. Details of sex, housing, and procedures using animals can be found in the Supplementary Information.

Amphetamine self-administration experiments were performed according to established methods [24, 25] with minor adjustments for mice. OCT3 mRNA expression in dopaminergic neurons in midbrain slices was assessed via multiplex fluorescence in situ hybridization (FISH) using RNAscope probes against TH and OCT3 (Cat No. 317621 and 439051-C2, Advanced Cell Diagnostics Inc., Newark, CA, USA) according to manufacturer instructions. In vivo high-speed chronoamperometry was utilized to measure amphetamine-evoked dopamine release and exogenous dopamine reuptake as performed previously [3, 18]. Immunohistochemistry to assess DAT expression was performed using primary antibodies against DAT (MAB369, Sigma Aldrich, St Louis, MO, USA) and Alexa Fluor 488 secondary antibodies (Cat #A-11006, ThermoFisher Scientific, Waltham, MA, USA). Detailed descriptions of all methods can be found in the Supplementary Information.

## RESULTS

### Amphetamine self-administration is dramatically attenuated in constitutive OCT3 knockout mice

**Fig. 1:**
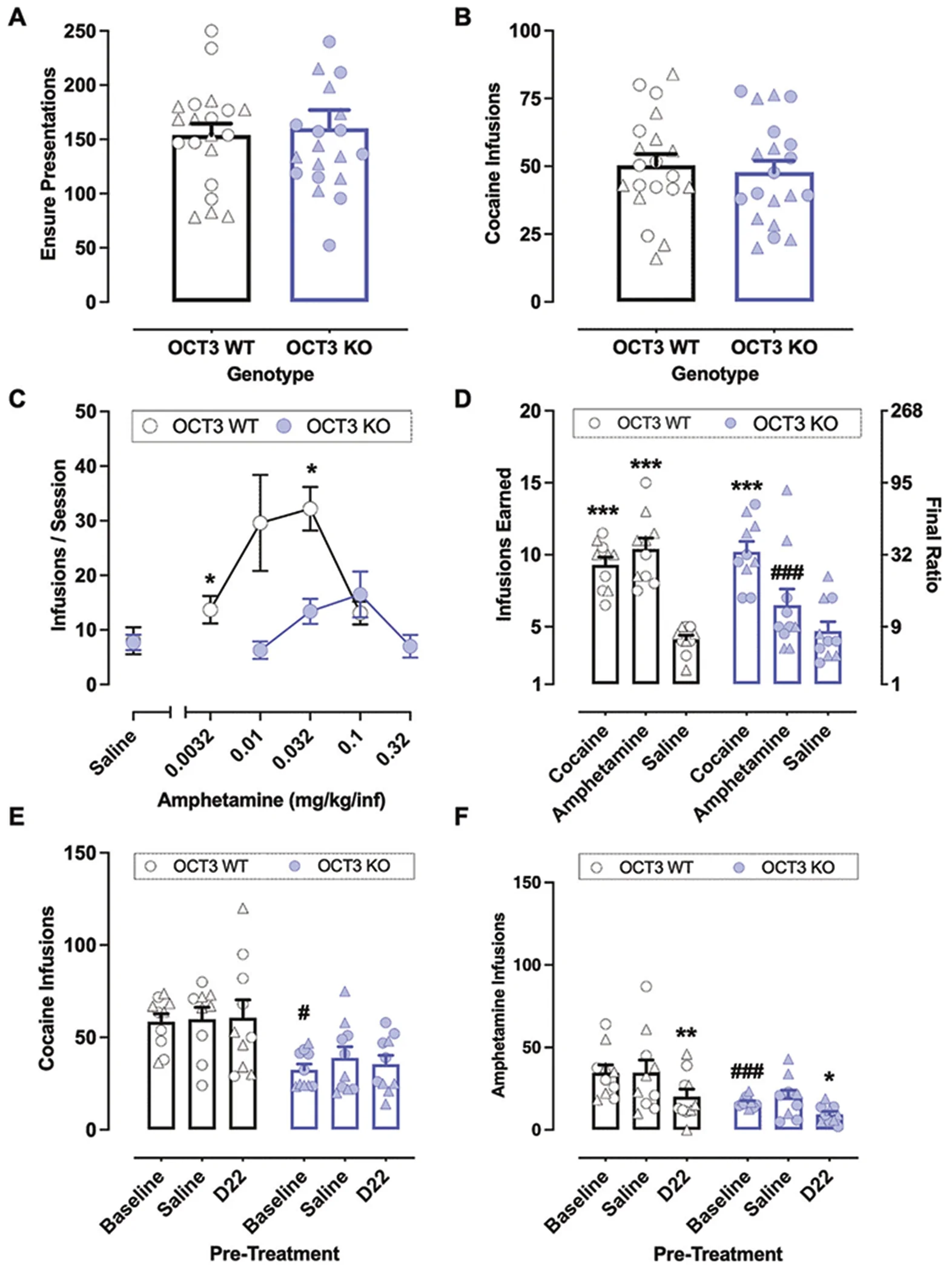
Amphetamine self-administration is attenuated in constitutive OCT3 knockout mice. **A** Responding for food in wildtype and OCT3 knockout mice. Bars are mean and SEM. Circles are males and triangles are females. Responding for food did not differ between wildtype and OCT3 knockout mice under an FR1 schedule of reinforcement. **B** Cocaine (0.32 mg/kg/infusion) self-administration in wildtype and OCT3 knockout mice. Bars are mean and SEM. Circles are males and triangles are females. Self-administration of cocaine did not differ between wildtype and OCT3 knockout mice on an FR1 schedule of reinforcement. **C** Amphetamine (0.0032–0.32 mg/kg/infusion) self-administration in wildtype and OCT3 knockout mice on FR1 schedule of reinforcement. Data points represent mean and SEM. OCT3 knockout mice exhibit attenuated amphetamine-self administration relative to wildtypes (Dose: *p* < 0.01; Genotype: *p* < 0.001, * *p* < 0.05 versus genotype-matched saline group). **D** Cocaine (0.32 mg/kg/infusion) and amphetamine (0.1 mg/kg/infusion) self-administration on PR schedule of reinforcement in wildtype and OCT3 knockout mice. Bars are mean and SEM. Circles are males and triangles are females. Cocaine self-administration did not differ between wildtype and OCT3 knockout mice under a PR schedule of reinforcement; however, OCT3 knockout mice responded at lower levels for amphetamine than wildtypes (Reinforcer: F(2,18) = 25.05, *p* < 0.0001; Genotype: not significant; Interaction F(2,18) = 9.33, *p* < 0.01; *** *p* < 0.01 versus saline within genotype; ### *p* < 0.001 versus wildtype within reinforcer). **E** Effect of D22 pre-treatment on cocaine (0.32 mg/kg/infusion) self-administration under an FR1 schedule of reinforcement in wildtype and OCT3 knockout mice. Bars are mean and SEM. Circles are males and triangles are females. There was a main effect of genotype with cocaine self-administration being lower in OCT3 knockout mice relative to wildtypes (F(1,9) = 13.98, *p* < 0.05; # *p* < 0.05 versus wildtype baseline group), but pre-treatment with D22 did not impact cocaine self-administration in either genotype. **F** Effect of D22 pre-treatment on amphetamine (0.032 mg/kg/infusion) self-administration under an FR1 schedule of reinforcement in wildtype and OCT3 knockout mice. Bars are mean and SEM. Circles are males and triangles are females. There was a main effect of genotype with amphetamine self-administration being lower in OCT3 knockout mice relative to wildtypes (F(1,9) = 7.71 *p* < 0.05; ### *p* < 0.001 versus wildtype baseline group); there was a main effect of pre-treatment in which D22 attenuated amphetamine self-administration in both wildtype and OCT3 knockout mice (F(2,18) = 13.31, *p* < 0.001 * *p* < 0.05, ** *p* < 0.01 versus genotype-matched baseline group). **A** and **B**: *n* = 20 males and 20 females. **C–F**: *n* = 10 males and 10 females. There were no significant sex effects so data for males and females are pooled.

Previous findings indicate a role for OCT3 in development of amphetamine CPP, as amphetamine CPP was attenuated in wildtype mice by inhibition of OCT3 with D22, and did not develop in constitutive OCT3 knockouts [19]. However, these data examined only one dose of amphetamine. Amphetamine CPP typically produces an inverted U-shaped dose-effect relationship [26–28]. Thus, it is unclear whether the attenuation in CPP observed by inhibition of OCT3 is consistent with a decrease or increase in rewarding effects (i.e., a rightward or leftward shift in the dose-effect curve). To clarify this, we first sought to characterize contributions of OCT3 to the reinforcing properties of amphetamine using IVSA in wildtype and constitutive OCT3 knockout mice. We found that constitutive OCT3 knockouts showed no difference from wildtypes in responding for food (Fig. 1A) or self-administration of cocaine (0.32 mg/kg/infusion) (Fig. 1B). However, OCT3 knockouts earned significantly fewer infusions of amphetamine under a fixed ratio (FR) 1 schedule of reinforcement over a range of doses compared to their wildtype counterparts (Fig. 1C). Amphetamine self-administration did not significantly differ from saline self-administration in OCT3 knockout mice at any of the doses tested (Fig. 1C). Under a progressive ratio (PR) schedule of reinforcement, OCT3 knockouts responded at wildtype levels for cocaine, but at lower levels for amphetamine (Fig. 1D). Inhibition of OCT3 with D22 had no effect on cocaine self-administration under PR in either genotype (Fig. 1E) but decreased responding for amphetamine in wildtype mice (Fig. 1F). D22 also attenuated amphetamine self-administration in OCT3 knockouts (Fig. 1F), though this effect was modest. Importantly, this dose of D22 does not produce aversive [19] or sedative [3] effects that could impact performance of the task. These data suggest that another D22-sensitive transporter in addition to OCT3 may also be involved in the reinforcing effects of amphetamine. Consistent with this notion, we have previously shown that PMAT may play a role in rewarding effects of amphetamine [19].

### Generation of conditional OCT3 knockdown mice

**Fig. 2:**
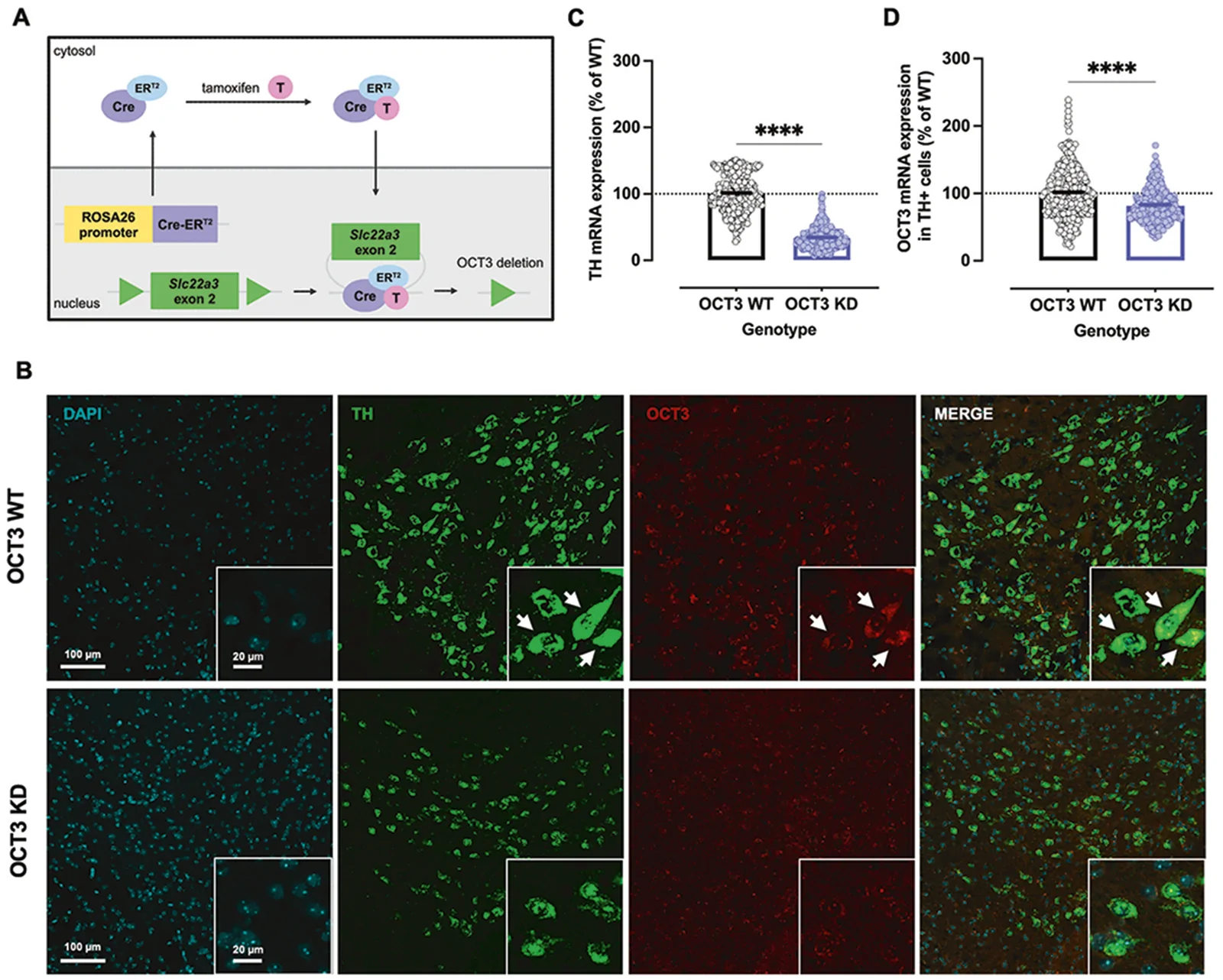
Generation of tamoxifen-inducible global OCT3 knockdown mice. **A** Schematic of tamoxifen-inducible global OCT3 knockdown mouse design. Tamoxifen-inducible Cre-ER^T2^ is expressed under the control of ROSA26 promoter, enabling ubiquitous expression. The ER^T2^ moiety retains Cre recombinase in cytoplasm until binding to tamoxifen. Tamoxifen allows translocation of the Cre recombinase complex to the nucleus and permits recombination at loxP sites flanking exon 2 of the *Slc22a3* gene encoding for OCT3. **B** Expression of OCT3 in tyrosine hydroxylase (TH)-positive cells in VTA. Multiplex fluorescent in situ hybridization (FISH) using RNAscope probes against *TH* (green) and *OCT3* (red) was performed on adult fixed-frozen tissue sections. Arrows indicate colocalization of TH and OCT3, illustrating expression of OCT3 in VTA dopamine neurons in OCT3 wildtype mice. OCT3 mRNA expression is reduced in tamoxifen-treated OCT3 knockdown mice (bottom) relative to tamoxifen-treated wildtype mice (top). **C** Quantification of TH mRNA expression in VTA. Bars are mean and SEM. TH mRNA levels are reduced in OCT3 knockdown mice (two-tailed Mann-Whitney test, **** *p* < 0.0001). **D** Quantification of OCT3 mRNA expression in TH-positive cells in VTA. Bars are mean and SEM. OCT3 mRNA in midbrain TH-positive cells is reduced in OCT3 knockdown mice (two-tailed Mann-Whitney test, **** *p* < 0.0001).

Our self-administration data (Fig. 1) and published findings using constitutive OCT3 knockouts suggest a role for OCT3 in neurochemical and behavioral actions of amphetamine [3, 19]. Next, we generated temporally inducible OCT3 knockdown mice to corroborate these results, eliminating confounds of developmental differences or compensation of other transporters. OCT3 floxed mice (OCT3^fl/fl^) were generated and crossed with mice expressing a tamoxifen-inducible Cre-ER^T2^ moiety under the expression of the ROSA26 promoter (R26^Cre^ mice). Crossing of these lines yields R26^Cre^:OCT3^fl/fl^ mice exhibiting tamoxifen- inducible global OCT3 knockdown (Fig. 2A). Using RNAscope, we confirmed expression of OCT3 in midbrain dopamine neurons of wildtypes (Fig. 2B) and validated approximately 20% knockdown of OCT3 mRNA in midbrain dopamine neurons of tamoxifen- treated OCT3 knockdowns (Fig. 2D). Tyrosine hydroxylase (TH) expression was also reduced in OCT3 knockdown mice (Fig. 2C), consistent with reports of reduced tissue levels of dopamine in constitutive OCT3 knockouts [29]. OCT3 knockdown mice were viable and showed no locomotor deficits in open field (Supplementary Fig. S1A), although they did demonstrate increased exploratory behavior (Supplementary Fig. S1B), consistent with reports of reduced anxiety-like behavior in constitutive OCT3 knockouts [30].

### OCT3 contributes to amphetamine-evoked dopamine release in dorsal striatum

**Fig. 3:**
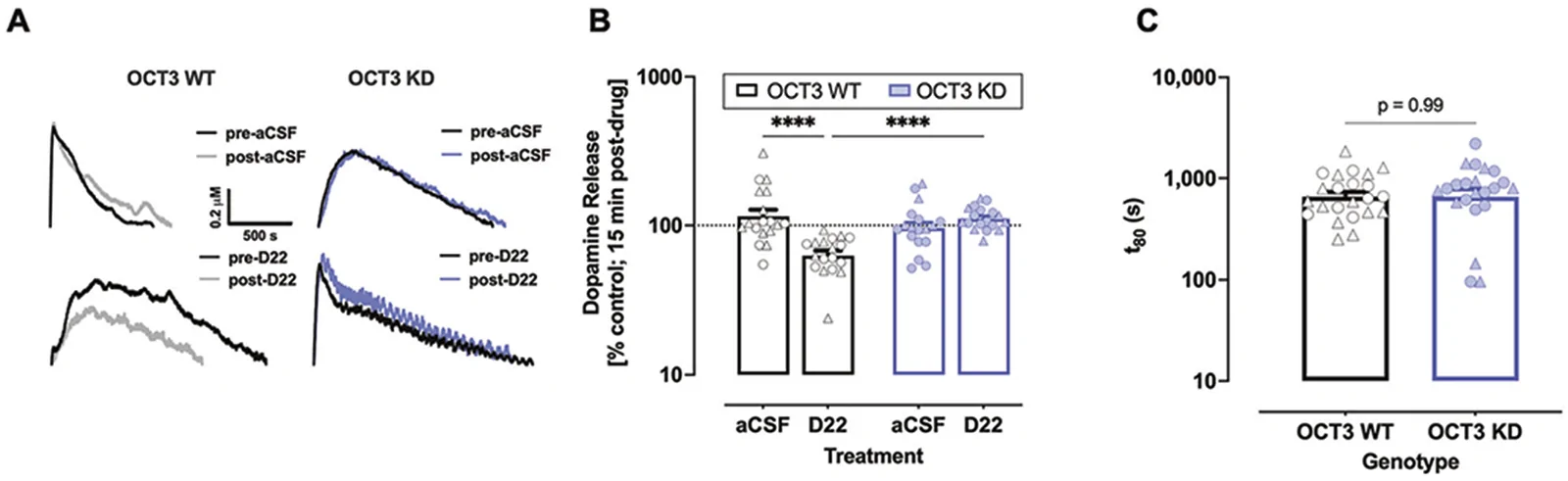
Inhibition of OCT3 attenuates amphetamine-evoked dopamine release, but not clearance, in dorsal striatum. **A** Representative oxidation signals produced by amphetamine in dorsal striatum. Release was found to be primarily dopamine based on ratios of reduction to oxidation currents ( > 0.45, Supplementary Table S1). Black traces are amphetamine (50 pmol)-evoked dopamine release at baseline; Gray and purple traces are amphetamine-evoked dopamine release 15 min after administration of vehicle (artificial cerebrospinal fluid, aCSF) or D22 (1 pmol) in wildtype and OCT3 knockdown mice, respectively. Post-drug traces are superimposed on baseline traces for ease of comparison. **B** Effect of aCSF and D22 on peak amplitude of amphetamine-evoked dopamine release in dorsal striatum (see also Supplementary Fig S3 for sexes separated and Supplementary Tables S2–S3) 15 min post administration of drug. Bars are mean and SEM. Circles are males and triangles are females. D22 attenuated amphetamine-evoked dopamine release in wildtype mice but not OCT3 knockdown mice (data log transformed to maintain normality, analyzed by two-factor mixed-effects analysis [treatment x genotype: F(1,68) = 22.68, *p* < 0.0001], followed by Sidak’s multiple comparisons test, **** *p* < 0.0001, *n* = 18 from 7–12 males and 6–11 females). Effects did not vary by sex (data log transformed to maintain normality, analyzed by three-factor mixed-effects analysis [treatment x genotype x sex: F(1,64) = 0.03, *p* = 0.86). **C** Baseline amphetamine-evoked dopamine clearance times (t_80_) in dorsal striatum of wildtype and OCT3 knockdown mice. Bars are mean and SEM. Circles are males and triangles are females. t_80_ values did not differ between genotypes (data log transformed to maintain normality, unpaired t-test, t(43) = 0.01, *p* = 0.99). t_80_ values did not differ by sex (data log transformed to maintain normality, two-way ANOVA [sex: F(1,41) = 0.81, *p* = 0.37; genotype x sex: F(1,41) = 0.13, *p* = 0.71]). *n* = 22–23 from 10 to 13 males and 9–13 females.

We have previously shown that D22 attenuates amphetamine-evoked dopamine release in dorsal striatum, an effect lost in constitutive OCT3 knockouts [3]. Here, we used in vivo high-speed chronoamperometry to replicate these findings in tamoxifen-inducible OCT3 knockdown mice (Fig. 3A–C). We found no differences in baseline amphetamine-evoked dopamine release between wildtype and OCT3 knockdown mice (Supplementary Table S1), consistent with constitutive OCT3 knockouts [3]. However, baseline amphetamine-evoked dopamine release varied by sex. Female mice exhibited lesser amphetamine-evoked dopamine release in dorsal striatum at baseline relative to males, regardless of genotype (Supplementary Fig. S2). Inhibition of OCT3 with D22 attenuated amphetamine-evoked dopamine release in dorsal striatum of wildtype mice 15 min post-drug (Fig. 3A, B; Supplementary Tables S2 and S3). In contrast, D22 had no effect on amphetamine-evoked dopamine release in dorsal striatum of OCT3 knockdown mice (Fig. 3A, B; Supplementary Tables S2 and S3), confirming specificity of D22 effects at OCT3. Importantly, despite marked reductions in TH expression in OCT3 knockdown mice (Fig. 2C), baseline amphetamine-evoked dopamine release did not differ from wildtypes; therefore, the loss of D22 effect in OCT3 knockdown mice is not confounded by changes in dopamine availability. Rather, these data are consistent with OCT3 contributing to amphetamine-evoked dopamine efflux in dorsal striatum. The effect of D22 in wildtype mice had not completely dissipated 60 minutes post-drug (Supplementary Fig. S3) but was peak at the 15-minute timepoint. The effect of D22 to attenuate amphetamine-evoked dopamine release was maintained in R26^Cre^:OCT3^fl/fl^ mice that were not treated with tamoxifen to induce knockdown of OCT3 (Supplementary Fig. S4), ensuring the lack of effect of D22 in OCT3 knockdown mice is not a result of insertion of Cre recombinase into the genome.

### OCT3 does not significantly contribute to clearance of dopamine in dorsal striatum

Clearance of amphetamine-evoked dopamine was assessed by evaluating the time for peak dopamine amplitude to decay by 80% (t_80_). We found that amphetamine-evoked dopamine t_80_ values in dorsal striatum did not differ between wildtype and OCT3 knockdown mice at baseline (Fig. 3C). Evidence from our lab [18], and others [15, 17], have previously suggested that OCT3 may contribute to dopamine clearance in striatum. It is possible that the negligible impact of OCT3 knockdown on amphetamine-evoked dopamine clearance in striatum was due to the presence of amphetamine, in which OCT3 may be primarily mediating dopamine efflux rather than clearance. Additionally, OCT3 is believed to contribute to clearance of extracellular monoamines when high-affinity transport mechanisms (i.e., DAT) are saturated or pharmacologically compromised [31]. Thus, it is possible that the concentration of dopamine released in amphetamine-evoked dopamine experiments (~0.5μM) was not sufficiently high to engage OCT3. Thus, we sought to characterize the extent to which OCT3 may contribute to dopamine clearance in dorsal striatum under drug-naïve conditions at varying dopamine concentrations.

**Fig. 4:**
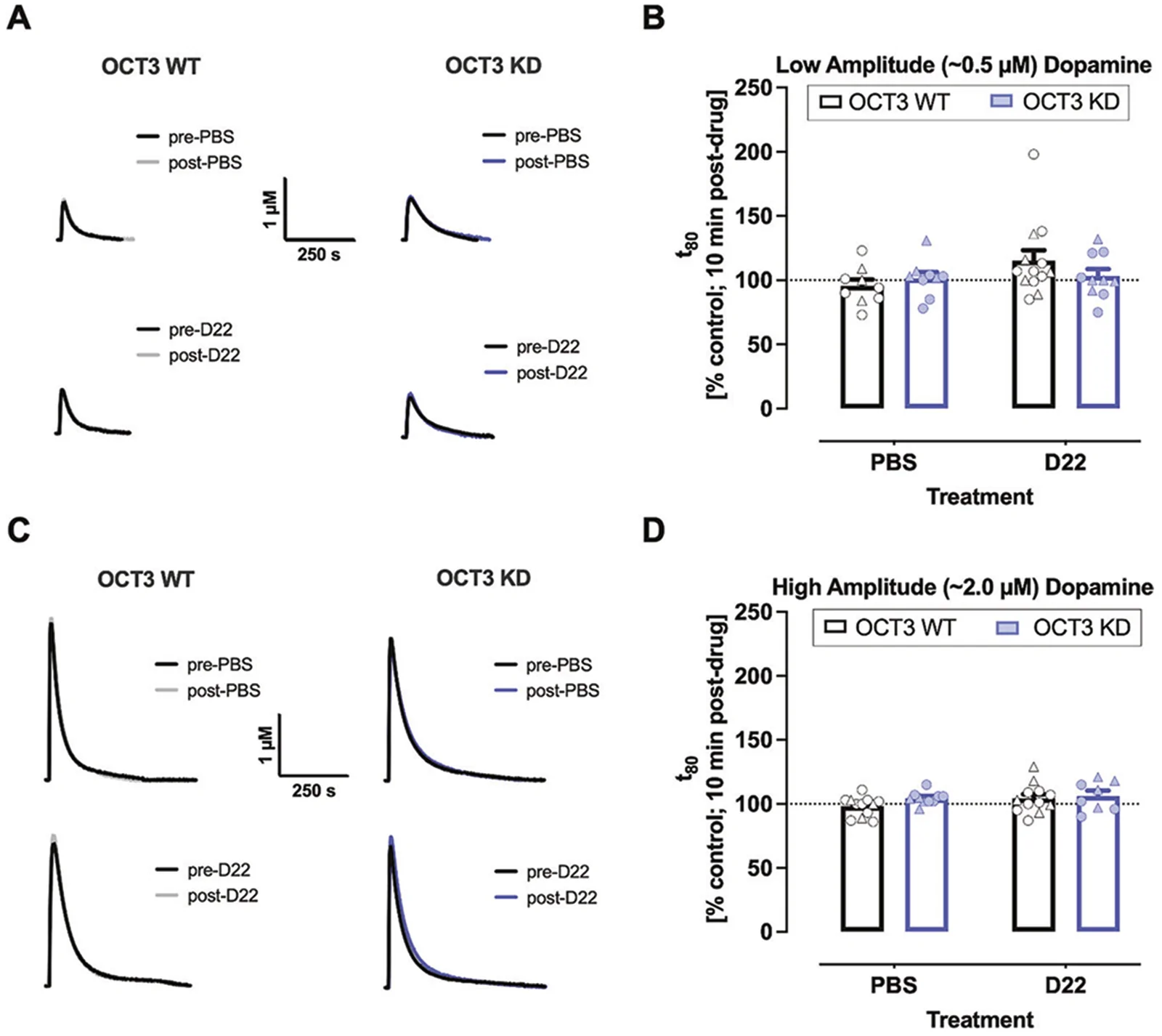
Inhibition of OCT3 failed to attenuate exogenous dopamine clearance in dorsal striatum. **A** Representative low amplitude oxidation signals produced by pressure ejection of dopamine (0.57 ± 0.01 μM, ~8.5 pmol in 43 nL) in dorsal striatum. Black traces are baseline dopamine signals; Gray and purple traces are dopamine signals 10 min after administration of vehicle (phosphate buffered saline, PBS) or D22 (1 pmol) in wildtype and OCT3 knockdown mice, respectively. Post-drug traces are superimposed on baseline traces for ease of comparison. **B** Effect of PBS or D22 on low amplitude dopamine (0.57 ± 0.01 μM, ~8.5 pmol in 43 nL) t_80_ clearance times 10 min post administration of drug. Bars are mean and SEM. Circles are males and triangles are females. Percent change in t_80_ values did not differ by genotype nor treatment (two-factor mixed effects analysis [treatment: F(1,37) = 2.57, *p* = 0.12; genotype: F(1,37) = 0.21, *p* = 0.65; treatment x genotype: F(1,37) = 1.80, *p* = 0.19]). Percent change in t_80_ values did not differ by sex (three-factor mixed effects analysis [sex: F(1,33) = 0.23, *p* = 0.64; treatment x sex: F(1,33) = 0.89, *p* = 0.35; genotype x sex: F(1,33) = 0.84, *p* = 0.37; treatment x genotype x sex: F(1,33) = 0.0026, *p* = 0.96]). *n* = 9–13 from 5–8 males and 3–5 females. **C** Representative high amplitude oxidation signals produced by pressure ejection of dopamine (2.29 ± 0.05 μM, ~10.6 pmol in 53 nL) in dorsal striatum. Black traces are baseline dopamine signals; Gray and purple traces are dopamine signals 10 min after administration of vehicle (PBS) or D22 (1 pmol) in wildtype and OCT3 knockdown mice, respectively. Post-drug traces are superimposed on baseline traces for ease of comparison. D Effect of PBS or D22 on high amplitude dopamine (2.29 ± 0.05 μM, ~10.6 pmol in 53 nL) t_80_ clearance times 10 min post administration of drug. Bars are mean and SEM. Circles are males and triangles are females. Percent change in t_80_ values did not differ by genotype nor treatment (two-factor mixed effects analysis [treatment: F(1,13) = 2.42, *p* = 0.14; genotype: F(1,25) = 2.14, *p* = 0.16; treatment x genotype: F(1,13) = 1.01, *p* = 0.33]). A minor sex difference was observed (three-factor mixed-effects analysis [treatment x sex: F(1,11) = 5.61, *p* = 0.037]), but no post-hoc comparisons achieved statistical significance (Sidak’s multiple comparisons test, *p* > 0.38). *n* = 8–13 from 4 to 9 males and 3–5 females.

To characterize the contributions of OCT3 to dopamine clearance in dorsal striatum, we again used in vivo high-speed chronoamperometry to assess the impact of D22 on clearance of dopamine exogenously applied to dorsal striatum of wildtype and OCT3 knockdown mice. We examined effects of D22 at a “low” concentration of dopamine (to achieve a signal amplitude of 0.57 ± 0.01 µM), which is within the range of affinity for DAT to be primarily responsible for dopamine uptake [32], and a “high” concentration of dopamine (to achieve a signal amplitude of 2.29 ± 0.05 μM), which is thought to exceed the capacity of DAT and has previously been shown to be sufficient to engage OCT3 [31]. There were no significant differences in baseline exogenous dopamine clearance parameters between wildtype and OCT3 knockdown mice (Supplementary Table S4). D22 did not prolong clearance of exogenous dopamine at low or high amplitudes of dopamine, regardless of genotype (Fig. 4A–D; Supplementary Table S4). These data are consistent with the lack of effect of OCT3 knockdown on clearance of amphetamine-evoked dopamine in dorsal striatum (Fig. 3C) and suggest that inhibition of OCT3 may make it possible to selectively attenuate amphetamine-evoked dopamine release when amphetamine is taken without disrupting homeostasis of dopamine clearance and prolonging its effects in the extracellular milieu.

### Contributions of OCT3 to amphetamine-evoked dopamine release may be striatal subregion-dependent

**Fig. 5:**
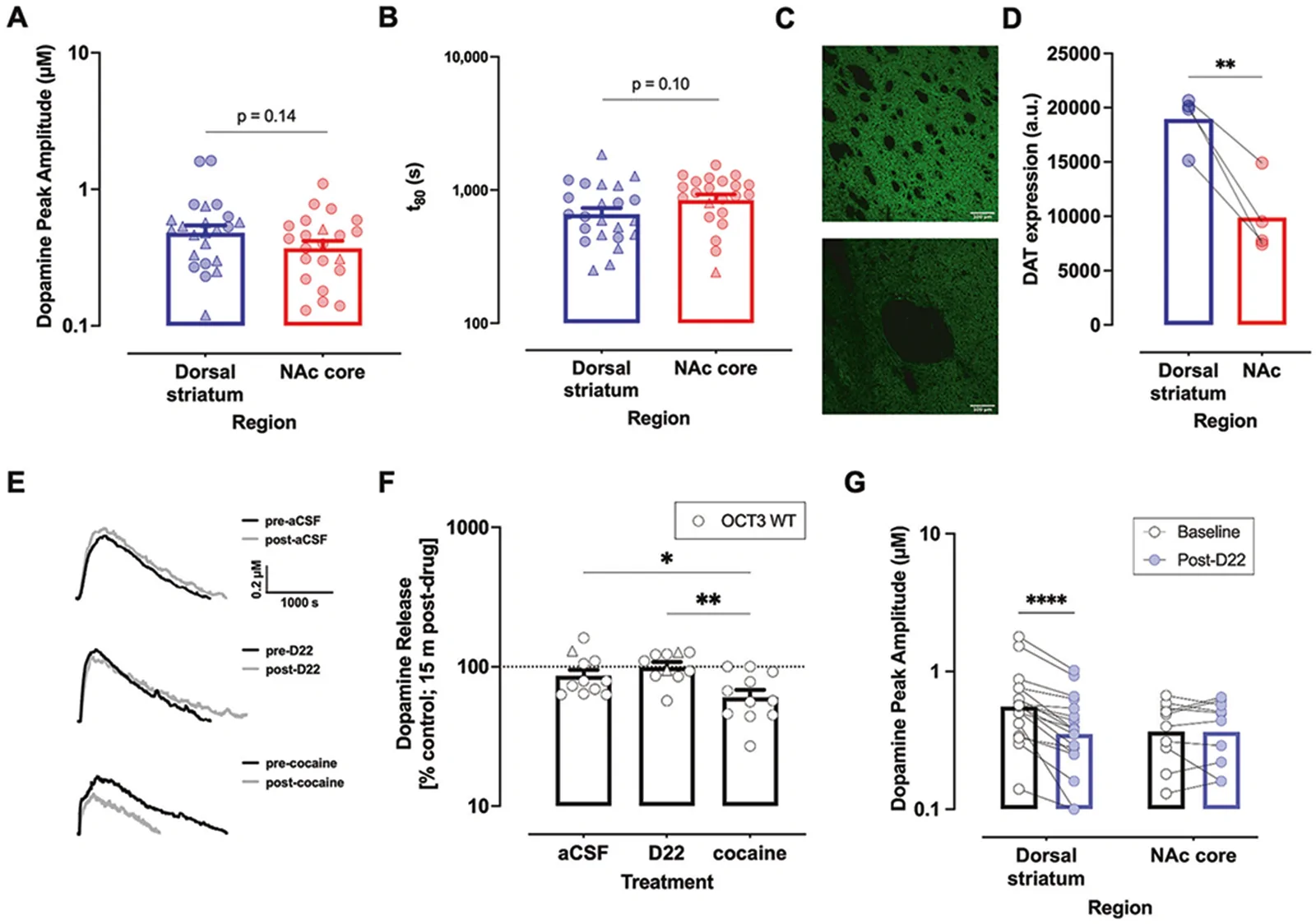
Inhibition of OCT3 failed to attenuate amphetamine-evoked dopamine release in nucleus accumbens core. **A** Baseline amphetamine (50pmol)-evoked dopamine release peak amplitude between dorsal striatum and nucleus accumbens core of wildtype mice. Bars are mean and SEM. Circles are males and triangles are females. Data were log transformed to maintain normality. Peak amplitudes did not differ between dorsal striatum and nucleus accumbens core (unpaired t-test, t(43) = 1.51, *p* = 0.14). **B** Baseline amphetamine-evoked dopamine clearance times (t_80_) in dorsal striatum and nucleus accumbens core of wildtype mice. Bars are mean and SEM. Circles are males and triangles are females. t_80_ values did not differ between dorsal striatum and nucleus accumbens core (unpaired t-test, t(43) = 1.68, *p* = 0.10). **C** Expression of DAT in dorsal striatum (top) and nucleus accumbens core (bottom). Expression of DAT is lower in nucleus accumbens core relative to dorsal striatum. **D** Quantification of DAT expression in dorsal striatum and nucleus accumbens core. Bars are mean and SEM. DAT expression is lower in nucleus accumbens core relative to dorsal striatum (paired t-test, t(3) = 5.86, ** *p* = 0.0099). **E** Representative oxidation signals produced by amphetamine in nucleus accumbens core. Release was found to be primarily dopamine based on ratios of reduction to oxidation currents ( > 0.45, Supplementary Table S4). Black traces are amphetamine (50 pmol)-evoked dopamine release at baseline; Gray traces are amphetamine-evoked dopamine release 15 min after administration of vehicles (aCSF), D22 (1 pmol), or cocaine (80 pmol) in wildtype mice. Post-drug traces are superimposed on baseline traces for ease of comparison. **F** Effect of aCSF, D22, and cocaine on peak amplitude of amphetamine-evoked dopamine release in nucleus accumbens core (see also Supplementary Tables S5–S6) 15 min post administration of drug. Bars are mean and SEM. Circles are males and triangles are females. D22 failed to attenuate amphetamine-evoked dopamine release in wildtype mice, but cocaine robustly attenuated amphetamine-evoked dopamine release in nucleus accumbens (data log transformed to maintain normality, one-way ANOVA [F(2,29) = 6.30, *p* = 0.0053], followed by Tukey’s multiple comparisons test, * *p* < 0.05, ** *p* < 0.01). *n* = 10–11 from 8 to 11 males and 0–2 females. **G** Comparison of effect of D22 on amphetamine-evoked dopamine release in wildtype mice in dorsal striatum versus nucleus accumbens core. Bars represent the mean. Baseline amphetamine-evoked dopamine release is plotted in black, and the respective amphetamine-evoked dopamine release following D22 is plotted in purple. D22 robustly attenuated amphetamine-evoked dopamine release in wildtype mice in dorsal striatum, but not nucleus accumbens core (two-way repeated measures ANOVA [region x treatment: F(1,24) = 10.01, *p* = 0.0042], followed by Sidak’s multiple comparisons test, **** *p* < 0.0001).

After confirming our previous findings indicating a role for OCT3 in amphetamine-evoked dopamine release in dorsal striatum, we sought to understand if OCT3 similarly contributes to amphetamine-evoked dopamine release in NAc. Thus, we evaluated the effect of D22 on amphetamine-evoked dopamine release in NAc core using in vivo high-speed chronoamperometry. We did not find any significant differences in baseline amphetamine-evoked dopamine release and clearance parameters between wildtype mice in NAc core relative to dorsal striatum (Fig. 5A, B; Supplementary Table S5), although peak amplitude was modestly lower and clearance trended to be longer in NAc core. This is consistent with reduced DAT expression in NAc core relative to dorsal striatum (Fig. 5C, D). Contrary to our findings in dorsal striatum, inhibition of OCT3 with D22, at the concentration tested, did not attenuate amphetamine-evoked dopamine release in NAc core of wildtype mice (Fig. 5E, F; Supplementary Tables S6 and S7). We were unable to test higher concentrations of D22 due to interference with the electrode at higher concentrations. However, inhibition of DAT with cocaine robustly attenuated amphetamine-evoked dopamine release in NAc core of wildtype mice 15 min post-drug (Fig. 5E, F; Supplementary Tables S6 and S7). This effect had diminished by 60 min post-drug (Supplementary Fig. S5). The robust effect of D22 to attenuate amphetamine-evoked dopamine release in dorsal striatum but not in NAc core (Fig. 5G) raises the possibility that contributions of OCT3 to amphetamine-evoked dopamine release may be striatal subregion-dependent.

## DISCUSSION

The most significant finding of this study is that OCT3 plays a major role in mediating neurochemical and abuse-related effects of amphetamine. Behaviorally, we show that pharmacological or genetic inhibition of OCT3 attenuates amphetamine IVSA, without impacting responding for food or cocaine. Then, we developed a tamoxifen-inducible OCT3 knockout model and replicated our findings in dorsal striatum illustrating OCT3’s contributions to amphetamine-stimulated dopamine efflux. While inhibition of OCT3 attenuated dopamine release in dorsal striatum, it did not significantly impact dopamine clearance, suggesting OCT3 can be selectively targeted to attenuate amphetamine-stimulated dopamine release without impacting dopamine homeostasis, a critically important characteristic for development of therapeutics for amphetamine-type stimulant use disorders. Lastly, OCT3 inhibition, at the dose of D22 tested, did not significantly impact amphetamine-induced dopamine release in NAc core, indicating its role in the actions of amphetamine may be subregiondependent. Altogether, these data suggest OCT3 may serve as a novel target for treatment of amphetamine-type stimulant use disorder.

First, we used IVSA in constitutive OCT3 knockout mice to build off previous findings indicating a role for OCT3 in behavioral (amphetamine stimulated locomotor activity [3] and CPP [19]) effects of amphetamine and amphetamine-evoked dopamine release [3]. Previous studies examined one dose of amphetamine [19], making it difficult to discern the directional effect of OCT3 knockout on amphetamine reward. Here, we utilized multiple doses to characterize the contribution of OCT3 to reinforcing properties of amphetamine. We found attenuated amphetamine self-administration in constitutive OCT3 knockout mice compared to wildtypes under both FR and PR schedules. Further, amphetamine self-administration did not significantly differ from saline in OCT3 knockouts under either schedule of reinforcement. These data suggest OCT3 is critical to reinforcing properties of amphetamine. Considering DAT knockouts still exhibit amphetamine-evoked dopamine release and CPP [12, 13], these findings indicate OCT3 may be as important as DAT in mediating amphetamine reinforcement. Importantly, OCT3 knockout also had no effect on cocaine nor food self-administration, indicating effects are specific to OCT3’s role in amphetamine’s actions. These results highlight the potential for targeting OCT3 for treatment of amphetamine-type stimulant use disorder.

The lack of effect of OCT3 knockout on cocaine selfadministration was unexpected, considering one would expect combined inhibition of DAT and OCT3 to slow dopamine clearance more than inhibition of DAT alone. Previous studies have determined a role for corticosterone-mediated inhibition of OCT3 driving the stress-induced potentiation of cocaine-primed reinstatement of conditioned reward [15, 16], driven by corticosterone-mediated prolongation of dopamine clearance in NAc hypothesized to be mediated via inhibition of OCT3 [15]. However, they found no differences in development of CPP between wildtype and OCT3 knockouts [16]. Considering this, data from the present study illustrating no impact of pharmacological or genetic inhibition of OCT3 on dopamine clearance in dorsal striatum, and published data showing no impact of OCT3 inhibition on dopamine clearance in NAc [17], it is likely that any effect of OCT3 knockout on dopamine clearance was insufficient to impact reinforcing effects of cocaine.

We found no effect of OCT3 inhibition on dopamine clearance in dorsal striatum. This lack of effect may be due to partial knockdown of OCT3 or partial occupancy of D22 at OCT3, considering we have previously shown a modest effect of constitutive OCT3 knockout to slow dopamine clearance in dorsal striatum [33]. We could not test higher concentrations of D22 in these experiments due to interference with the recording electrode at higher doses. Despite this, this dose of D22 was sufficient to produce marked changes in amphetamine-evoked dopamine release in dorsal striatum. We conclude that contributions of OCT3 to dorsal striatum dopamine clearance are minimal in comparison to much larger observed contributions to amphetamine-evoked dopamine release. In line with this, other groups have only shown contributions of OCT3 to striatal dopamine clearance when DAT is concurrently inhibited [15, 17]. In contrast, we have shown that ethanol, which acutely inhibits OCT3, prolongs dopamine clearance in dorsal striatum [33]. The concentration of ethanol was not limited due to methodological constraints, as the concentration of D22 was here. Therefore, at higher doses of OCT3 inhibitors, we have shown an effect of OCT3 inhibition on striatal dopamine clearance. Further, inhibition of OCT3 with ethanol augmented the ability of cocaine to inhibit dopamine clearance in dorsal striatum [33]. Thus, as we [33] and others [15, 17] have shown, it is possible that OCT3 inhibition may slow striatal dopamine clearance when DAT is dually inhibited or when larger concentrations of OCT3 inhibitor are used; however, these data suggest contributions of OCT3 alone to dopamine clearance in striatum are minimal, especially in comparison to contributions to amphetamine-evoked dopamine efflux, and may be negligible when the full complement of DAT is intact.

The observed lack of effect of OCT3 inhibition on dopamine clearance is a desirable quality in a drug target for the treatment of amphetamine-type stimulant use disorder. Specifically attenuating amphetamine-evoked dopamine efflux via OCT3 inhibition without prolonging effects of dopamine in extracellular milieu allows for attenuation of reinforcement without disrupting normal physiology of the dopamine system. This is important, as normal functioning of the dopamine system is essential to maintaining proper regulation of motivation, motor control, cognition, and mood. Further, polysubstance use is common among drug users [34]; were a therapeutic used to treat amphetamine-type stimulant misuse also to cause prolonged dopamine clearance, it could potentiate reinforcing effects of other substances. Thus, OCT3 is an attractive target for therapeutic development, considering its specificity to the mechanism of action of amphetamine-type stimulants.

Another unsuspected finding of the present study was that D22 pre-treatment attenuated amphetamine self-administration in OCT3 knockouts. These data point towards involvement of an additional D22-sensitive, OCT3-independent mechanism in mediating amphetamine reinforcement. We have previously shown that PMAT may also be involved in amphetamine reward [19]. Thus, it is possible that the ability of D22 pre-treatment to maintain inhibition of amphetamine self-administration in OCT3 knockouts may be mediated by D22 effects at PMAT. Further studies are required to evaluate this.

After characterizing the contributions of OCT3 to amphetamine self-administration, we generated temporally inducible OCT3 knockout mice to validate our findings indicating a role for OCT3 in amphetamine-evoked dopamine release in dorsal striatum. Using these mice, we replicated our findings that inhibition of OCT3 in dorsal striatum attenuates amphetamine- evoked dopamine release, an effect lost in OCT3 constitutive knockout [3] and conditional knockout mice. We observed a complete loss of D22 effects in conditional OCT3 knockout mice here despite only modest knockdown of OCT3. It is likely that if a higher, near-maximal concentration of D22 were used, there would be only a partial loss of D22 effect, due to contributions of residual OCT3 or potential effects of D22 at PMAT, as seen in behavioral experiments. Altogether, these data support our working model that amphetamine induces dopamine efflux at OCT3 in addition to DAT. Validation of these experiments using the temporally-inducible OCT3 knockout mice eliminates potential confounds of compensation or developmental differences associated with use of constitutive knockouts, strengthening model validity.

We extended these experiments to evaluate contributions of OCT3 to neurochemical actions of amphetamine in NAc core. Although we found no significant differences in amphetamine- stimulated dopamine release and reuptake between regions, peak dopamine amplitude and clearance time in NAc core trended to be lower and longer relative to dorsal striatum. This is consistent with our data and literature indicating higher DAT expression and differential dopamine dynamics in dorsal versus ventral striatum [35, 36]. We found no effect of OCT3 inhibition on amphetamine- evoked dopamine release in NAc core, in contrast to its ability to robustly attenuate amphetamine-evoked dopamine release in dorsal striatum. This finding indicates that contributions of OCT3 to neurochemical actions of amphetamine may be striatal subregion-dependent. Further, these subregion differences indicate that behavioral differences in OCT3 knockout mice may be linked to neurochemical differences in dorsal rather than ventral striatum, the latter of which is more famously associated with reward learning and reinforcement. Although the dorsal striatum is canonically associated with locomotion and habit formation, it has been implicated in reward [37, 38]. These data support this notion and highlight that roles of dorsal and ventral striatum may not be as functionally distinct as previously thought.

Despite the lack of effect of D22 on amphetamine-evoked dopamine release in NAc core observed, we cannot rule out the possibility of OCT3 contributing to neurochemical effects of amphetamine in NAc. It is likely the role of OCT3 in amphetamine-evoked dopamine release in NAc may be dose-dependent. As the present study evaluated one dose of amphetamine and one dose of D22, one cannot rule out the possibility that OCT3 could be involved in mediating effects of amphetamine in NAc. Additional experiments evaluating multiple doses of amphetamine will be necessary. Further, although the dose of D22 cannot be raised due to interference at higher doses with the recording electrode, it would be informative to evaluate amphetamine-evoked dopamine release in NAc with full OCT3 knockout. Thus, we cannot rule out the possibility that OCT3 contributes to amphetamine’s action in NAc to some extent; however, contributions of OCT3 to neurochemical actions of amphetamine are more prominent in dorsal striatum under these conditions. This is consistent with literature indicating potentially higher expression of OCT3 in dorsal striatum relative to NAc core [39].

Altogether, data presented here indicate OCT3 plays an important role in mediating neurochemical and behavioral actions of amphetamine, making OCT3 an attractive candidate for development of therapeutics to treat amphetamine-type stimulant use disorder. Despite the promise for OCT3 as a novel therapeutic target, much work is needed before its potential can be evaluated clinically. A major hurdle for research is a lack of safe, selective ligands to target OCT3. Efforts have been made to synthesize selective inhibitors [40–43], but much work is needed on this front. Medicinal chemistry to develop a selective OCT3 inhibitor will be essential to advance this work. Further, OCT3 is acutely inhibited by corticosterone in rodents and cortisol in humans, which are induced by amphetamine, and therefore may complicate this interaction and impact efficacy of a potential OCT3 inhibitor. More research is required to evaluate this possibility when considering OCT3 as a potential target for amphetamine- type stimulant use disorder.

## Supplementary Material

Supplementary material

The online version contains supplementary material available at https://doi.org/10.1038/s41386-026-02390-6.

## Data Availability

Data is available upon request.
