# Supplementary material for: It’s not all DAT: harnessing the potential of organic cation transporter 3 inhibition to selectively attenuate amphetamine reinforcement and dopamine release
Source: Neuropsychopharmacology. Author manuscript; Available in PMC 2026 May 27. (PMC13215722; doi:10.1038/s41386-026-02390-6)
Supplement: Supplementary material [file NIHMS2164489-supplement-Supplementary_material.docx]

**Supporting Information for**

**It’s not all DAT: Harnessing the potential of organic cation transporter 3 inhibition to selectively attenuate amphetamine reinforcement and dopamine release**

^†^Lauren E. Honan^1,2^, Ph.D.; ^†^Briana Mason^2^, Ph.D.; William Anthony Owens^1^, M.S.; Sangbin Shin^1^, B.S.; Yeon Ha Ju^3,4^, Ph.D.; Yonggong Shi^2^, M.D.; Rebecca E. Horton^1^, B.S.; Lief E. Fenno^3,4^, Ph.D.; *Gregory T. Collins^2,5^, Ph.D.; *Lynette C. Daws^1,2^, Ph.D.

^†^These authors contributed equally to this work

^1^Department of Cellular & Integrative Physiology, University of Texas Health Science Center at San Antonio, San Antonio, TX, 78229, USA

^2^Department of Pharmacology, University of Texas Health Science Center at San Antonio, San Antonio, TX, 78229, USA

^3^Department of Neuroscience, University of Texas at Austin, Austin, TX, 78712, USA

^4^Department of Psychiatry and Behavioral Sciences, Dell Medical School at UT Austin, Austin, TX, 78712, USA

^5^South Texas Veterans Health Care System, San Antonio, TX, 78229, USA

*Corresponding authors:

Gregory T. Collins

University of Texas Health Science Center at San Antonio

Department of Pharmacology

7703 Floyd Curl Drive, MC7764

San Antonio, TX, 78229-3900, USA

Ph: (210) 567-4199

Email: [collinsg@uthscsa.edu](mailto:collinsg@uthscsa.edu)

Lynette C. Daws

University of Texas Health Science Center at San Antonio

Departments of Cellular & Integrative Physiology & Pharmacology

7703 Floyd Curl Drive, MC7756

San Antonio, TX, 78229-3900, USA

Ph: (210) 567-4361

Email: [daws@uthscsa.edu](mailto:daws@uthscsa.edu)

**DETAILED METHODS**

**Animals**

Naïve adult (>P90) male and female wildtype (OCT3 WT) and constitutive OCT3 knockout (OCT3 KO) mice (originally developed by (1)) were obtained from an in-house colony at the University of Texas Health Science Center at San Antonio (UTHSCSA) and were used for all self-administration experiments. OCT3 floxed (OCT3^fl/f38–40l^) mice were generated by the Mouse Genome Engineering and Transgenic Facility at UTHSCSA. A B6SJLF2 female was used as one of the two founders. B6SJLF2 mice are a second-generation hybrid mouse strain (C57BL/6J x SLJ/J), which are commonly used as host embryos for microinjecting transgenic constructs to create transgenic mice. Offspring were mated and backcrossed with C57BL/6J mice. The other founder female was C57BL/6J. OCT3^fl/fl^ mice were crossed with Gt(ROSA)26sortm^38–41^l(Cre/ERT2tyip)/J (R26^Cre^) mice (stock #008463, Jackson Laboratory, Bar Harbor, ME, USA, originating article: (2)) expressing Cre recombinase ubiquitously to generate an in-house colony of R26^Cre^:OCT3^fl/fl^ mice with tamoxifen inducible global OCT3 knockdown. Adult (>P90) male and female OCT3^fl/fl^ (OCT3 WT) and R26^Cre^:OCT3^fl/fl^ (OCT3 knockdown, KD) mice were used for RNAscope, open field test, and *in vivo* high-speed chronoamperometry experiments in dorsal striatum. Adult (>P90) male and female OCT3^fl/fl^ mice were used immunohistochemistry and for viral injections and subsequent *in vivo* high-speed chronoamperometry experiments in nucleus accumbens core. All mice were bred on a C57BL/6 background, housed in a temperature-controlled (24°C) vivarium on a 12/12-hour light/dark cycle (lights on at 07:00 h), and were provided ad libitum access to food and water. All procedures were conducted in accordance with an approved Institutional Animal Care and Use Committee (IACUC) protocol and abided by current NIH guidelines.

To achieve at least 80% power, sample sizes were estimated (G*Power) from the smallest effect sizes considered to be important based on our previous publications (3,4). Both male and female mice were used in all analyses, but studies were not adequately powered to detect sex differences as this was not the focus of the study. Thus, analyses were performed with sexes pooled to assess genotype and treatment effects, which were the focus of this research, and are reported in the main figures. In most cases, data were assessed for sex effects as a secondary outcome if applicable, which are reported in the figure captions if relevant and are to be followed up on in separate studies.

**Intravenous Self-Administration**

Adult wildtype (n=10 male, 10 female) and constitutive OCT3 knockout mice (n=10 male, 10 female) were singly housed with ad libitum water and ~5g daily standard chow per day. Experiments used standard operant chambers (Med Associates, St. Albans, VT) with two retractable levers, yellow LEDs above each lever, and a houselight on the opposite wall. A liquid dipper provided access to a 10 μL cup via a recessed food trough between levers. External variable speed syringe pumps delivered infusions through tygon tubing connected to fluid swivels and spring tethers held by counterbalance arms.

All mice were initially trained to respond for 15-sec presentations of 10 μL chocolate Ensure (50% in water) during daily 90-min sessions under an FR1 schedule of reinforcement. Illumination of the LED above the active lever (left/right; counterbalanced) signaled reinforcer availability. Dipper presentation was paired with a 15-sec illumination of an LED above the food cup; responding during this period and all Inactive lever responses were recorded but had no consequences. Acquisition of responding for food was defined as >15 food presentations over three consecutive sessions in which stability criteria were met (≥80% active lever responses and <20% day-to-day reinforcer variation).

After food training, mice were anesthetized with 3% isoflurane and surgically implanted with indwelling left femoral vein catheters attached to vascular access buttons externalized in the mid-scapular region. Post-surgery, mice received Exceed (20 mg/kg *s.c.*) to prevent infection and meloxicam (1 mg/kg *s.c.*) for analgesia. A 7-day recovery period was provided, during which catheters were flushed daily with 0.2 mL heparinized saline (50 U/mL).

For intravenous self-administration, catheters were flushed with 0.2 mL saline before the session and mice were allowed to lever press for delivery of cocaine (0.32 mg/kg/inf) during daily 90-min FR1 sessions. Infusions were paired with a 5-sec houselight illumination during which time responses were recorded but had no consequences. Acquisition of responding for cocaine was defined as >15 infusions over three consecutive sessions that also met stability criteria. After acquisition, subsets [n=10 wildtype (5M, 5F), 10 OCT3 knockout (5M, 5F) per experiment] were used to: 1) generate amphetamine dose-response curves, or 2) evaluate pretreatments with D22, and responding under a progressive ratio (PR) schedule.

Amphetamine dose-response curves were generated by substituting various doses of amphetamine (0.0032-0.32 mg/kg/inf) or saline for cocaine for at least 5 sessions and until responding was stable. In between doses of amphetamine (or saline), mice were allowed to respond for cocaine so that all doses were substituted from a similar baseline.

Effects of D22 (0.1 mg/kg *i.p.*) and saline were evaluated as 30-min pretreatments in mice responding stably for cocaine (0.32 mg/kg/inf) or amphetamine (0.1 mg/kg/inf) under an FR1 schedule. The order of reinforcer and treatment were counterbalanced across mice. Upon completion of D22 tests, mice were allowed to respond for cocaine (0.32 mg/kg/inf) under a PR schedule of reinforcement. For PR testing, response requirements incremented per the equation: ratio=(5e^infusion#×0.2)−5, with sessions lasting at most 4 hr but terminating after 45 min without ratio completion (5,6). Mice initially responded for cocaine until stable (≤2 infusion variance over 2 sessions) followed by substitution with amphetamine (0.1 mg/kg/inf) and saline; mice returned to cocaine before testing the alternate reinforcer.

*Data Analysis:* Responding maintained by Ensure and cocaine were analyzed by two-factor ANOVA (sex and genotype). Dose-response curves for amphetamine were first analyzed by two-factor (sex and dose) to determine if self-administration varied by sex, after which data were collapsed across sex and reanalyzed by a mixed effects model (dose and genotype) followed by post-hoc Dunnett’s tests for multiple comparisons. Effects of D22 (treatment and genotype) and levels of responding under the PR (reinforcer and genotype) were analyzed by two-factor ANOVA with post-hoc Sidak’s tests for multiple comparisons.

**Tamoxifen Injections**

Adult (>P90) male and female OCT3^fl/fl^ and R26^Cre^:OCT3^fl/fl^ mice received four consecutive daily 70 mg/kg intraperitoneal (*i.p.)* injections of tamoxifen (Hello Bio Inc, Princeton, NJ, USA) dissolved in a 4:1 mixture of sunflower and castor oils to induce knockdown of OCT3. A subset of animals received only the sunflower and castor oil mixture as a control (Supplementary Figure S4). All experiments were begun 28 days following the start of injections.

**RNAscope**

*Assay:* Fixed-frozen tissue was used for RNAscope assays, and was prepared as follows. Male mice were anesthetized and intracardially perfused with ice-cold phosphate buffered saline (PBS), followed by ice-cold 4% paraformaldehyde (PFA). Brains were extracted and placed in 4% PFA overnight at 4°C. After 24 hours, brains were transferred to 10% sucrose for 24 hours, then 20% sucrose for 24 hours, then 30% sucrose for 24 hours. 10 µm coronal sections were collected containing ventral tegmental area (VTA) and mounted onto Superfrost Plus microscope slides (Thermo Fisher Scientific, Waltham, MA, USA, Cat. No. 12-055-15), then stored at -80°C.

Commercially available RNAscope Multiplex Fluorescent reagent V2 kits and probes (Advanced Cell Diagnostics, Hayward, CA, USA, Cat. No. 323110) were used to quantify mRNA expression according to the manufacturer’s instructions. RNAscope probes against *tyrosine hydroxylase (TH)* (Cat. No. 317621) and *Oct3/Slc22a3* (Cat. No. 439051-C2) were used to assess OCT3 mRNA expression in TH-positive dopaminergic neurons. Briefly, slides were pretreated with Protease Pretreat III for 30 minutes at 40°C. Then, hybridization was performed at 40°C for two hours in a HybEZ oven (Advanced Cell Diagnostics, Hayward, CA, USA). Following hybridization, a series of washing and amplification steps were performed before counterstaining with DAPI. Slides were mounted using ProLong Gold Antifade Mountant (Thermo Fisher Scientific, Waltham, MA, USA, Cat. No. P36930) and coverslipped with 24 mm × 50 mm microscope cover glass. Sections containing VTA were imaged using a Nikon A1R confocal microscope at 20X magnification. All images were obtained using identical settings for gain, laser, and pinhole size.

*Data Analysis:* Images of VTA were obtained for two technical replicates each from two OCT3 wildtype and three OCT3 knockdown mice. Regions of interest (ROIs) were defined by manually drawing boundaries surrounding TH^+^ cell areas using ImageJ FIJI software. Mean TH and OCT3 fluorescence intensity in each ROI was quantified and expressed as a percent of the wildtype group mean. TH mRNA expression and OCT3 mRNA expression in TH^+^ cells were compared between genotypes via two-tailed Mann-Whitney test using GraphPad Prism.

**Immunohistochemistry**

*Assay:* Male OCT3^fl/fl^ mice were anesthetized and intracardially perfused with ice-cold heparinized PBS, followed by ice-cold 4% PFA. Brains were extracted and placed in 4% PFA overnight at 4°C. After 24 hours, brains were transferred to 30% sucrose for 36 hours. 50 µm coronal sections were collected containing dorsal striatum and nucleus accumbens core. Sections were kept in PBS at 4°C until use for staining.

Sections were blocked in 0.3% Triton X-100 (Sigma Aldrich, St. Louis, MO, USA) in PBS containing 5% normal goat serum (NGS, Abcam, Cambridge, UK) for 1 hour at 37°C. Sections were then incubated overnight at 4°C in primary antibody: rat anti-DAT (1:500, MAB369, Sigma Aldrich, St. Louis, MO, USA) in 0.3% Triton X-100 in PBS containing 5% NGS. Sections were rinsed 4 times for 15 minutes in PBS at room temperature. Afterwards, sections were incubated in secondary antibody for 1 hour at room temperature: Alexa Fluor 488 goat anti-rat (1:500, A-11006, Thermo Fisher Scientific, Waltham, MA, USA) in 0.3% Triton X-100 in PBS containing 5% NGS. Sections were then washed 4 times for 15 minutes in PBS at room temperature. Finally, sections were mounted on slides using Fluoromount-G with DAPI (Thermo Fisher Scientific, Waltham, MA, USA). Sections containing the dorsal striatum and nucleus accumbens core were imaged using a Zeiss confocal microscope at 20X magnification. All images were obtained using identical settings for gain, laser, and pinhole size.

*Data Analysis:* Images of dorsal striatum and nucleus accumbens core were obtained for three technical replicates each from four mice and were analyzed using ImageJ FIJI software (version 2.1.0/1.53c). Technical replicates were averaged to represent mean fluorescence intensity of each region per mouse, and DAT expression between regions was assessed via paired t-test using GraphPad Prism.

**Open Field Test**

*Assay:* Locomotor activity in OCT3 knockdown mice was characterized in an open field. Open field test was conducted in a white acrylic apparatus (42 cm x 42 cm x 42 cm). Mice were placed in the center of the apparatus and their locomotor activity was recorded for 10 minutes.

*Data Analysis:* Locomotor activity was recorded and analyzed with AnyMaze software (v5.2, Stoelting Co., Wood Dale, IL, USA). Total distance traveled and percent of time spent in center versus edges of the apparatus were log transformed to maintain normality and analyzed via by unpaired t-test using GraphPad Prism. Two-way ANOVA was utilized to assess potential sex effects, which were not observed (n = 13-16, 7-10 males and 6 females).

***In vivo* High-Speed Chronoamperometry**

*In vivo* high-speed chronoamperometry was used to characterize transporter function in amphetamine-evoked dopamine release and exogenous dopamine clearance experiments. Methods were adapted from our previous publications (3,4).

*Carbon Fiber Microelectrode Fabrication, Calibration, and Assembly:* Carbon fiber microelectrodes were fabricated with a 30 μm diameter carbon fiber (Specialty Materials, Lowell, MA, USA) sealed in fused silica (Schott, Rye Brook, NY, USA). Exposed carbon fiber was trimmed to 150 μm and coated with 5% Nafion (Sigma-Aldrich, St Louis, MO, USA) to enhance selectivity for dopamine over anionic metabolites. Recording electrodes were pre-calibrated *in vitro* for dopamine in the presence of its primary metabolite, 3,4 dihydroxyphenylacetic acid (DOPAC) as an interferant. Only those with greater than 100:1 selectivity for dopamine over DOPAC and linear responses (r^2^ > 0.9) were used. The recording electrode was fixed to a four-barrel glass micropipette (FHC, Bowdoinham, ME, USA) with the tips separated by approximately 200 μm. Barrels were filled with amphetamine (400 μM, Sigma-Aldrich, St Louis, MO, USA), D22 (10 μM, Sigma-Aldrich, St Louis, MO, USA), cocaine (800 µM, Sigma-Aldritch, St Louis, MO, USA), dopamine (200 µM), or vehicle (aCSF or saline, for amphetamine-evoked dopamine release experiments and exogenous dopamine clearance experiments, respectively). Note that due to diffusion the final concentration of solution that reaches the recording electrode is approximately 200-fold lower than the barrel concentration.

*High-Speed Chronoamperometric Recordings:* Animals were anesthetized with a urethane (250 mg/kg, Thermo Fisher Scientific, Waltham, MA, USA) and α-chloralose (25 mg/kg, Sigma-Aldrich, St Louis, MO, USA) mixture (*i.p.*), received tracheal intubation to facilitate breathing, and were secured in a stereotaxic apparatus (David Kopf Instruments, Tujunga, CA, USA). Body temperature was maintained at 36-37°C using a water circulated heating pad. The recording electrode/micropipette apparatus was lowered into dorsal striatum (+1.1 A/P, +1.4 M/L, -2.25 D/V from bregma/dura) or nucleus accumbens core (+0.8 A/P, +1.3 M/L, -4.0 D/V from bregma/dura). FAST-12 and FAST-16 systems (Quanteon, Nicholasville, KY, USA) were used to collect chronoamperometric recordings with the application of 100 ms +0.55 V oxidation potential pulses, separated by 900 ms intervals at 0 V resting potential, in respect to a Ag/AgCl reference electrode in the contralateral superficial cortex. Oxidation and reduction currents were averaged for the last 80 ms of each potential pulse. Exogenous drugs and neurotransmitter were pressure ejected into dorsal striatum using a Picospritzer II (General Valve Corporation, Fairfield, NJ, USA). For assessment of amphetamine-evoked dopamine release in dorsal striatum and nucleus accumbens core, amphetamine (50 pmol in 125 nL) was pressure-ejected to produce dopamine release ranging between ~0.1 to 2.0 μM. A period of 45 minutes was allotted between injections before making another replicate pressure-ejection of amphetamine. Then, D22 (1 pmol in 100 nL), cocaine (80 pmol in 100 nL), or an equivalent volume of vehicle (aCSF) was delivered. At 15 minutes and 60 minutes following, amphetamine was pressure-ejected again to compare signal amplitude to pre-drug replicates. For assessment of effects of D22 on exogenous dopamine clearance, reproducible dopamine signals were obtained at low (0.57 ± 0.01 µM, ~8.5 pmol in 43 nL) and high (2.29 ± 0.05 µM, ~10.6 pmol in 53 nL) concentrations. Once replicate dopamine signals were achieved, D22 (1 pmol in 100 nL) or an equivalent volume of vehicle (PBS) was delivered. Dopamine was pressure-ejected again every 10 minutes for 30 minutes. At the conclusion of experiments, an electrolytic lesion was made to mark electrode placement. Brains were extracted, frozen on dry ice, and stored at -80°C for histological verification.

*Data Analysis:* All statistical analyses were performed in GraphPad Prism. For amphetamine-evoked dopamine release experiments in dorsal striatum, peak amplitude data were expressed as a percent change from baseline and log transformed to maintain normality. Data were analyzed by two-factor (genotype, treatment) mixed-effects analysis followed by Sidak’s multiple comparisons test. Three-factor (genotype, treatment, sex) mixed-effects analysis was used to assess potential sex effects (n = 18 per group, 7-12 males and 6-11 females). For amphetamine-evoked dopamine release experiments in nucleus accumbens core, peak amplitude data were expressed as a percent change from baseline and log transformed to maintain normality. Data were analyzed by one-way ANOVA followed by Tukey’s multiple comparison test (n = 10-11 per group, 8-11 males and 0-2 females). For exogenous dopamine clearance experiments, t_80_ (time it takes peak amplitude to decay by 80%) data were analyzed by two-factor (genotype, treatment) mixed-effects analysis. Three-factor (genotype, treatment, sex) mixed-effects analysis was used to assess potential sex effects (n = 8-13 per group, 5-9 males and 3-6 females).

**Viral Injections**

OCT3^fl/fl^ mice used for amphetamine-evoked dopamine release experiments in nucleus accumbens core were injected bilaterally in VTA with an mCherry-labeling virus. Initial plans included an additional Cre-expressing virus group to assess specificity of D22 effects at OCT3 via loss of effect in mice with depletion of OCT3 from dopaminergic terminals in nucleus accumbens core; however, these experiments were not conducted, as no effect of D22 was observed in control animals. Thus, amphetamine-evoked dopamine release was only assessed in wildtype mice injected with the mCherry-labeling virus as detailed below.

OCT3^fl/fl^ mice were initially anesthetized using isoflurane (4%, Vet One Inc., Las Vegas, NV, USA), and anesthesia was maintained throughout the surgery at 1.0-2.5%. Ophthalmic ointment was applied to prevent eyes from drying and body temperature was maintained at 36-37°C using a circulating water heating pad. AAV5-Ef1a-mCherry (#114470-AAV5, Addgene, Watertown, MA, USA) was bilaterally injected in VTA (-3.3 A/P, +/-0.5 M/L, -4.1 D/V from bregma/dura) at a volume of 200 nL and a flow rate of 20 nL/min. Diffusion was allowed for 10 minutes after injection before removing the Hamilton syringe. Mice were then sutured, and treated with Enroflaxin (5 mg/kg, subcutaneous) and meloxicam (5 mg/kg, subcutaneous) post-operatively to prevent infection and alleviate pain. Chronoamperometric recordings were conducted 28 days post-surgery. Viral placement was evaluated after chronoamperometric recordings. Brains were post-fixed in 4% PFA overnight, sectioned at 50 µm, and mounted on slides with Fluoromount-G with DAPI. Viral placement in VTA was verified by mCherry fluorescence using a Keyence BZ-X710 All-in-One Fluorescence Microscope at 2X magnification.

SUPPLEMENTARY FIGURES

**A**

**B**


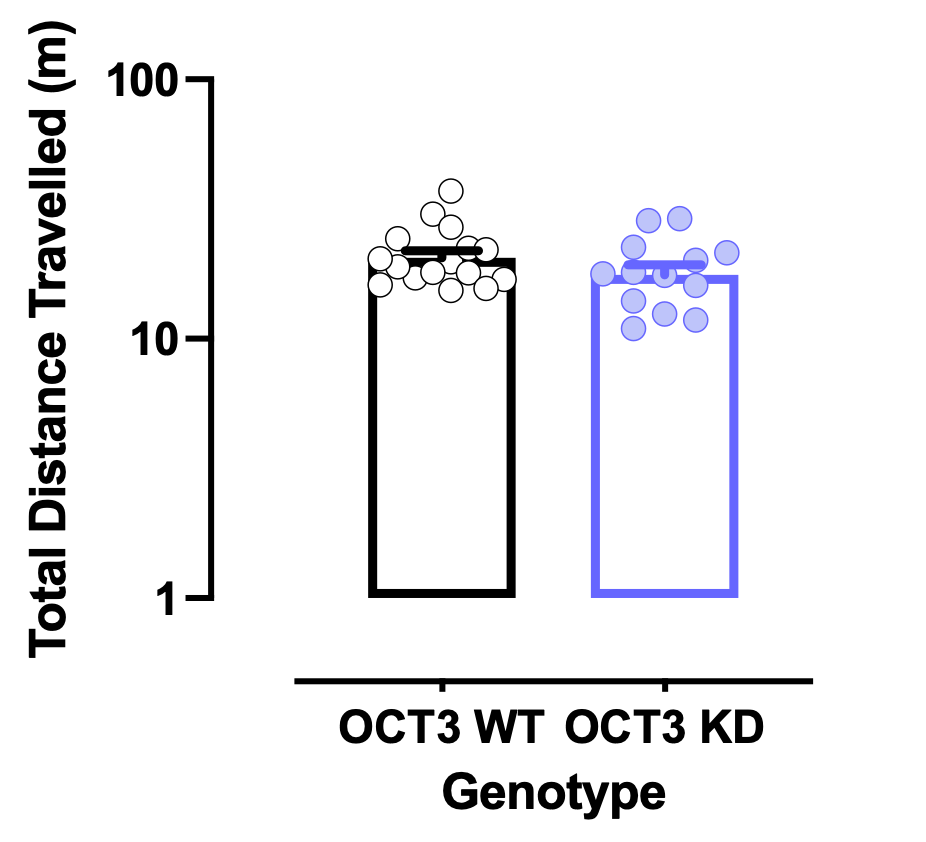

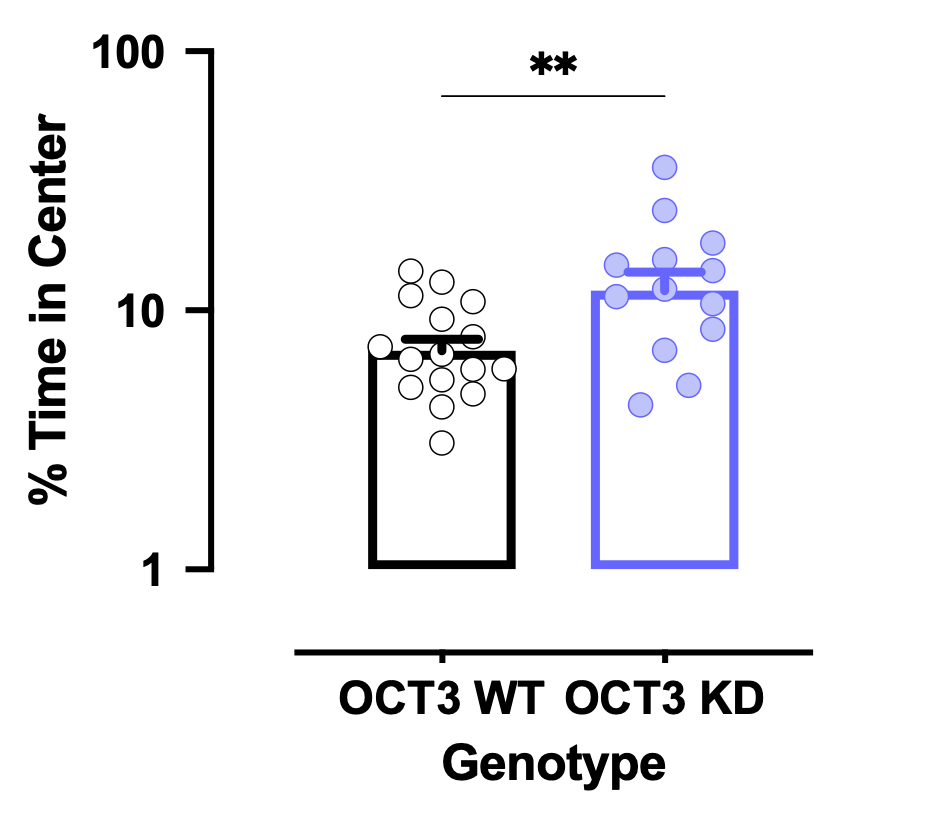


**Supplementary Figure S1.** OCT3 knockdown mice exhibit normal locomotor activity and enhanced exploratory behavior in open field. A) Total distance travelled in open field. Bars are mean and SEM. Distance travelled did not differ between wildtype and OCT3 knockdown mice (data log transformed to maintain normality, unpaired t-test, t(27) = 1.44, p = 0.16). Distance travelled did not vary by sex (two-way ANOVA, [sex: F(1,25) = 2.22, p = 0.15; genotype: F(1,25) = 4.48, p = 0.04; sex x genotype: F(1,25) = 1.60, p = 0.22]). B) Exploratory behavior in open field. Bars are mean and SEM. OCT3 knockdown mice spent more time in the center of the open field compared to wildtypes (data log transformed to maintain normality, unpaired t-test, t(27) = 2.85, ** p = 0.0082). Percent time in center of open field did not differ by sex (two-way ANOVA, [sex: F(1,25) = 0.96, p = 0.34; genotype: F(1,25) = 7.06, p = 0.01; sex x genotype: F(1,25) = 0.005, p = 0.95]). n = 13-16 from 7-10 males and 6 females.

**Supplementary Figure S2.** Female mice exhibit lesser amphetamine-evoked dopamine release in dorsal striatum. Baseline peak amplitude of amphetamine-evoked dopamine release in males and females. Bars are mean and SEM. Females exhibit lesser amphetamine-evoked dopamine release than male counterparts (data log transformed to maintain normality, unpaired t-test, t(43) = 2.18, * p = 0.035).


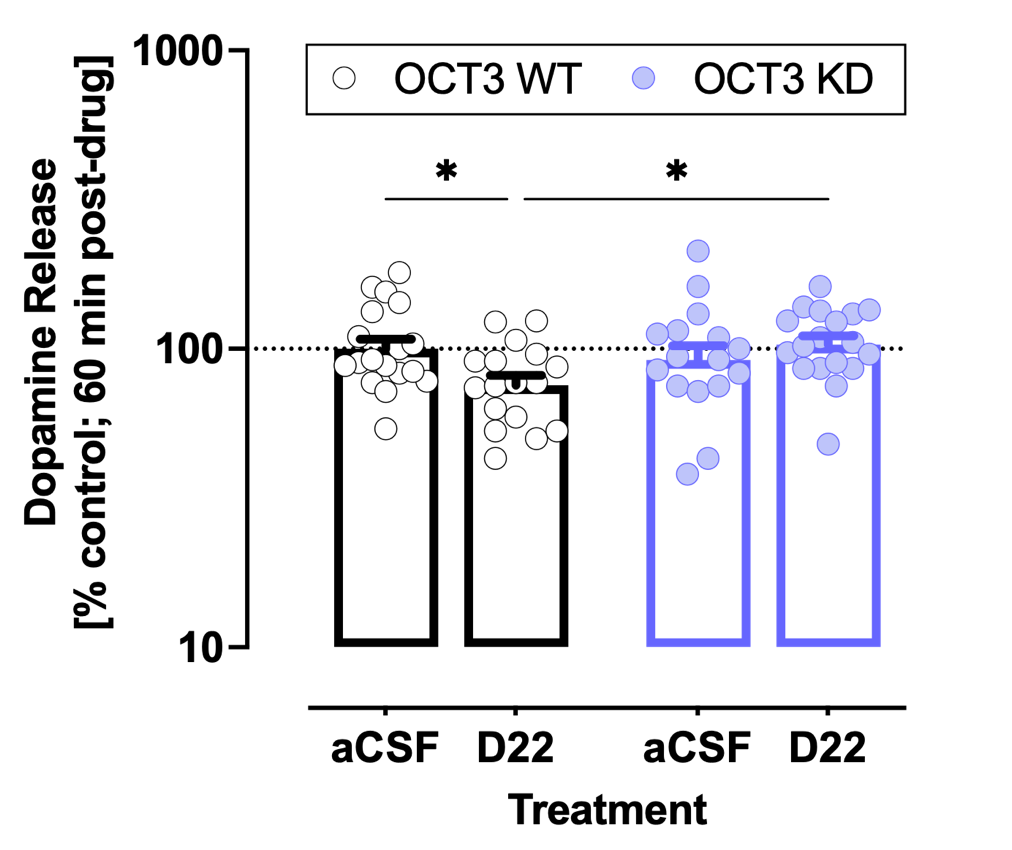


Supplementary Figure S3. D22 attenuates amphetamine-evoked dopamine release in dorsal striatum 60 minutes post-drug. Amphetamine-evoked dopamine release in dorsal striatum 60 minutes following drug administration. Bars are mean and SEM. D22 attenuated amphetamine-evoked dopamine release in dorsal striatum of wildtype mice, but not OCT3 knockdown mice (two factor mixed-effects analysis [treatment x genotype: F(1,65) = 6.02, p = 0.017], followed by Sidak’s multiple comparisons test, * p < 0.05). n = 16-18 from 7-12 males and 4-11 females. Effects did not differ by sex at 60 minutes post-drug (three-factor mixed-effects analysis [sex: F(1,61) = 1.90, p = 0.17; treatment x sex: F(1,61) = 0.54, p = 0.46; genotype x sex: F(1,61) = 0.02, p = 0.88; treatment x genotype x sex: F(1,61) = 1.17, p = 0.28]).


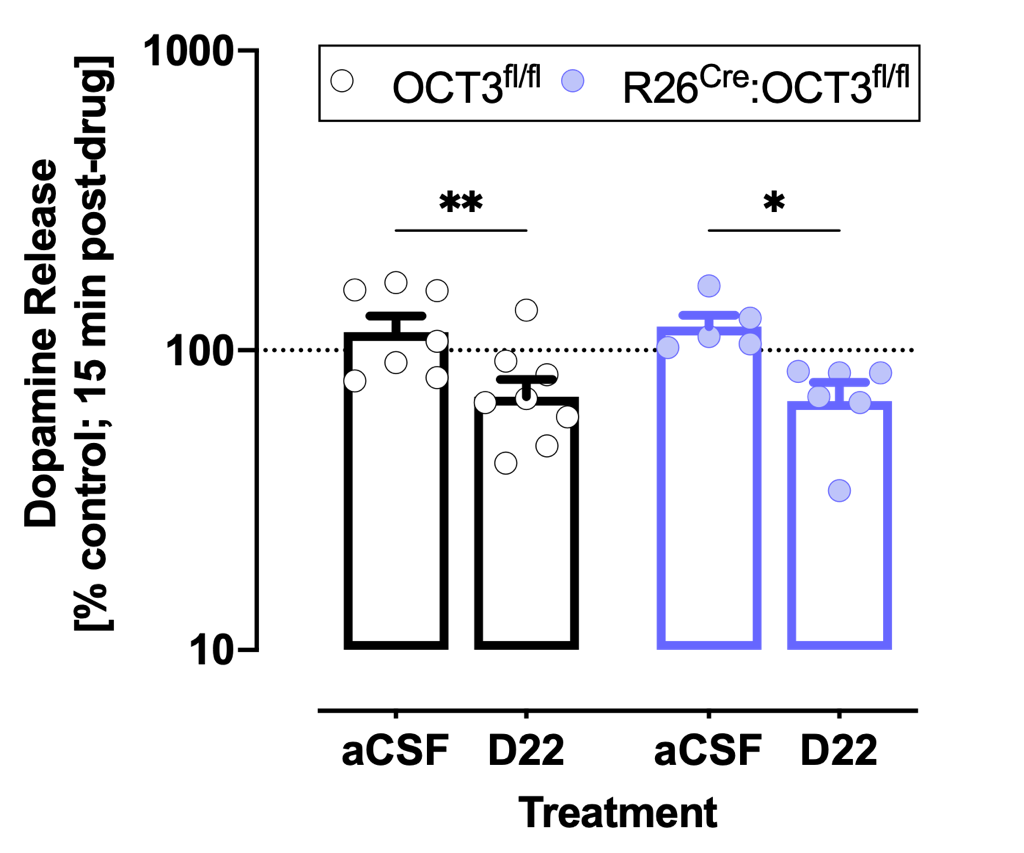


Supplementary Figure S4. D22 attenuates amphetamine-evoked dopamine release in dorsal striatum of oil-treated OCT3^fl/fl^ and R26^Cre^:OCT3^fl/fl^ mice. Amphetamine-evoked dopamine release in dorsal striatum 15 minutes following drug administration. Bars are mean and SEM. D22 attenuated amphetamine-evoked dopamine release in dorsal striatum of OCT3^fl/fl^ and R26^Cre^:OCT3^fl/fl^ mice treated only with oil as a control, not tamoxifen to induce knockdown (two factor mixed-effects analysis [treatment: F(1,9) = 23.15, p = 0.0010], followed by Sidak’s multiple comparisons test, * p < 0.05, ** p < 0.01). n = 5-8 from 1-6 males and 2-3 females. Sample sizes in this control experiment were not sufficiently powered to assess sex differences.


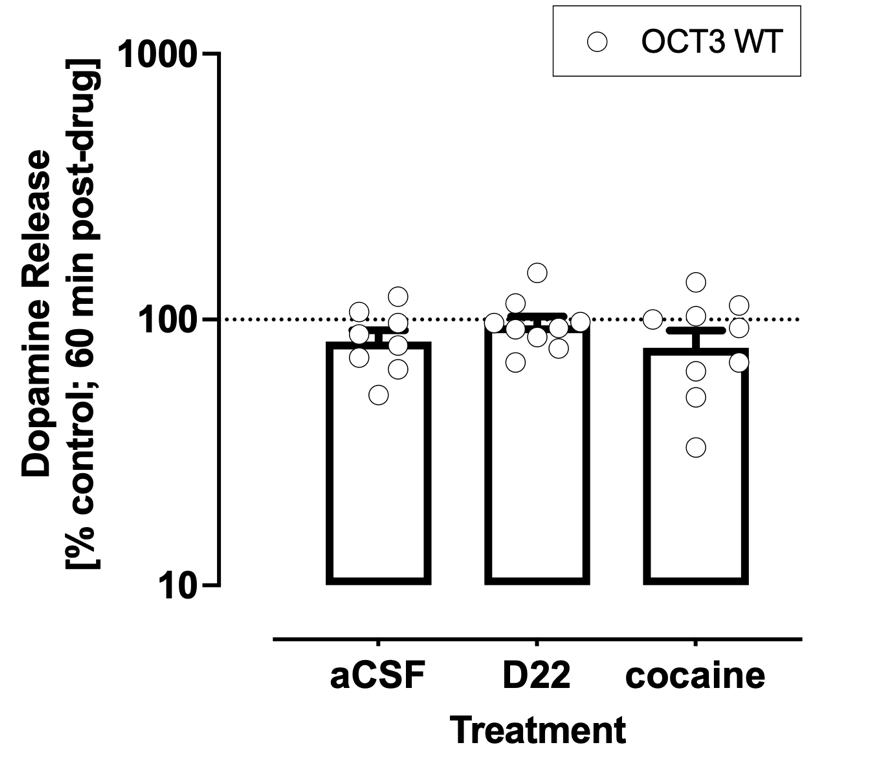


Supplementary Figure S5. Effect of cocaine to attenuate amphetamine-evoked dopamine release in nucleus accumbens core is diminished 60 minutes post-drug. Amphetamine-evoked dopamine release in nucleus accumbens 60 minutes following drug administration. Bars are mean and SEM. No significant effects of D22 nor cocaine on amphetamine-evoked dopamine release are observed in nucleus accumbens core 60 minutes following drug administration (one-way ANOVA [F(2,23) = 1.64, p = 0.45]). n = 8-9 from 8-9 males and 0-1 females.

SUPPLEMENTARY TABLES

**Supplementary Table S1. Comparison of baseline amphetamine-evoked dopamine release and clearance signal parameters in dorsal striatum of OCT3 wildtype and OCT3 knockdown mice.**

|  | **Peak Dopamine Release (µM)** | **Rise Time (s)** | **Clearance Time, t_80_ (s)** | **Clearance Rate (nM/s)** | **Red:Ox Ratio** |
| --- | --- | --- | --- | --- | --- |
| OCT3 WT  (n = 23) | 0.57 ± 0.08 | 214 ± 32 | 744 ± 80 | 1.0 ± 0.1 | 0.55 ± 0.03 |
| OCT3 KD  (n = 22) | 0.47 ± 0.07 | 180 ± 42 | 825 ± 101 | 0.8 ± 0.1 | 0.60 ± 0.02 |

There were no significant differences in signal parameters for baseline amphetamine-evoked dopamine release in dorsal striatum between OCT3 wildtype and OCT3 knockdown mice. Amphetamine-evoked release in dorsal striatum was primarily dopamine, as evidenced by reduction/oxidation current ratios being greater than 0.50. Data are mean and SEM. n = 22-23 from 10-13 males and 9-13 females.

**Supplementary Table S2. Effect of vehicle and D22 15 minutes post-infusion on amphetamine-evoked dopamine release and clearance signal parameters in dorsal striatum of OCT3 wildtype and OCT3 knockdown mice (percent change from baseline).**

|  | **aCSF** (n = 18) | **D22** (n = 18) |
| --- | --- | --- |
| **Peak Dopamine Release** | | |
| OCT3 WT  OCT3 KD | 127 ± 15  102 ± 9 | 66 ± 4 ****  113 ± 5 |
| **Rise Time** | | |
| OCT3 WT  OCT3 KD | 142 ± 41  148 ± 28 | 112 ± 12  151 ± 31 |
| **Clearance Time, t_80_** | | |
| OCT3 WT  OCT3 KD | 141 ± 20  115 ± 23 | 77 ± 9 **  131 ± 12 * |
| **Clearance Rate** | | |
| OCT3 WT  OCT3 KD | 91 ± 13  98 ± 11 | 153 ± 48  143 ± 71 |
| **Red:Ox Ratio** | | |
| OCT3 WT  OCT3 KD | 113 ± 8  119 ± 11 | 88 ± 9  91 ± 5 * |

The signal parameter most sensitive to the effect of D22 was peak dopamine release (see Figure 2 in main article and Supplementary Table S3). D22 significantly attenuated the peak amplitude of amphetamine-evoked dopamine release in dorsal striatum of wildtype mice, an effect not observed in OCT3 knockdown mice, confirming the specificity of effects at OCT3. Data are mean and SEM *percent change* from baseline amphetamine-evoked dopamine release signal (see Supplementary Table S3 for absolute values). * p < 0.05, ** p < 0.01, **** p < 0.0001, two-tailed Mann Whitney test compared to genotype-matched aCSF control group. n = 18 from 7-12 males and 6-11 females).

**Supplementary Table S3. Effect of vehicle and D22 15 minutes post-infusion on amphetamine-evoked dopamine release and clearance signal parameters in dorsal striatum of OCT3 wildtype and OCT3 knockdown mice (absolute change from baseline).**

|  | **aCSF** (n = 18) | | **D22** (n = 18) | |
| --- | --- | --- | --- | --- |
|  | **Pre-** | **Post-** | **Pre-** | **Post-** |
| **Peak Dopamine Release (µM)** | | | | |
| OCT3 WT  OCT3 KD | 0.55 ± 0.10  0.51 ± 0.08 | 0.58 ± 0.08  0.47 ± 0.06 | 0.65 ± 0.10  0.49 ± 0.08 | 0.42 ± 0.06 ****  0.55 ± 0.09 ** |
| **Rise Time (s)** | | | | |
| OCT3 WT  OCT3 KD | 222 ± 38  165 ± 25 | 236 ± 46  224 ± 60 | 184 ± 36  176 ± 52 | 173 ± 28  181 ± 37 |
| **Clearance Time, t_80_ (s)** | | | | |
| OCT3 WT  OCT3 KD | 696 ± 93  807 ± 115 | 837 ± 99  692 ± 72 | 856 ± 105  921 ± 131 | 619 ± 87 *  1159 ± 159 |
| **Clearance Rate (nM/s)** | | | | |
| OCT3 WT  OCT3 KD | - 1. ± 0.2   0.9 ± 0.2 | 0.8 ± 0.2  0.8 ± 0.1 | 1.0 ± 0.2  0.6 ± 0.1 | 1. ± 0.3   0.6 ± 0.1 * |
| **Red:Ox Ratio** | | | | |
| OCT3 WT  OCT3 KD | 0.53 ± 0.04  0.52 ± 0.04 | 0.57 ± 0.04  0.59 ± 0.05 | 0.58 ± 0.05  0.62 ± 0.03 | 0.51 ± 0.05  0.57 ± 0.04 |

The signal parameter most sensitive to the effect of D22 in dorsal striatum was peak dopamine release. D22 significantly attenuated the peak amplitude of dopamine release in wildtype mice (~0.23 µM decrease), an effect not observed in OCT3 knockdown mice. In fact, D22 had a minor effect to increase dopamine release in OCT3 knockdown mice (~0.06 µM increase), which is likely due to inhibitory effects on dopamine clearance via other D22-sensitive transporters (e.g., plasma membrane monoamine transporter). Data are mean and SEM. * p < 0.05, ** p < 0.01, **** p < 0.0001, two-tailed paired Mann-Whitney test comparing pre- versus post- signal parameter within genotype. n = 18 from 7-12 males and 6-11 females).

**Supplementary Table S4. Comparison of exogenous dopamine clearance parameters in dorsal striatum of OCT3 wildtype and knockdown mice.**

|  | | **Amplitude (µM)** | **Rise Time (s)** | **Clearance Time, t_80_ (s)** | **Clearance Rate (nM/s)** |
| --- | --- | --- | --- | --- | --- |
| OCT3 WT | Low dopamine  (n = 15) | 0.57 ± 0.02 | 14 ± 2 | 63 ± 15 | 17.7 ± 3.1 |
|  | High dopamine  (n = 16) | 2.27 ± 0.06 | 16 ± 2 | 82 ± 16 | 55.3 ± 12.9 |
| OCT3 KD | Low dopamine  (n = 11) | 0.57 ± 0.02 | 17 ± 2 | 81 ± 13 | 12.1 ± 2.8 |
|  | High dopamine  (n = 12) | 2.33 ± 0.09 | 15 ± 2 | 82 ± 10 | 38.4 ± 5.6 |

There were no significant differences in signal parameters for exogenous dopamine clearance between OCT3 wildtype and OCT3 knockdown mice in dorsal striatum. Data are mean and SEM. n = 11-16 from 5-9 males and 3-6 females.

**Supplementary Table S5. Comparison of baseline amphetamine-evoked dopamine release and clearance signal parameters in dorsal striatum versus nucleus accumbens core in wildtype mice.**

|  | **Peak Dopamine Release (µM)** | **Rise Time (s)** | **Clearance Time, t_80_ (s)** | **Clearance Rate (nM/s)** | **Red:Ox Ratio** |
| --- | --- | --- | --- | --- | --- |
| Dorsal striatum  (n = 23) | 0.57 ± 0.08 | 214 ± 32 | 744 ± 80 | 1.0 ± 0.1 | 0.55 ± 0.03 |
| Nucleus accumbens core  (n = 22) | 0.43 ± 0.05 | 268 ± 25 | 915 ± 71 | 0.5 ± 0.1 *** | 0.47 ± 0.03 |

Clearance rate was slower in nucleus accumbens core relative to dorsal striatum, consistent with lower expression of DAT (see **Figure 4** in main text). Data are mean and SEM. *** p < 0.001, two-tailed Mann-Whitney test comparing signal parameter in dorsal striatum and nucleus accumbens core.

**Supplementary Table S6. Effect of vehicle, D22, and cocaine 15 minutes post-infusion on amphetamine-evoked dopamine release and clearance signal parameters in nucleus accumbens core of OCT3 wildtype mice (percent change from baseline).**

|  | **aCSF** (n = 11) | **D22** (n = 10) | **Cocaine** (n = 11) |
| --- | --- | --- | --- |
| **Peak Dopamine Release** | | | |
| OCT3 WT | 91 ± 10 | 102 ± 7 | 65 ± 7 * |
| **Rise Time** | | | |
| OCT3 WT | 303 ± 205 | 126 ± 20 | 137 ± 19 |
| **Clearance Time, t_80_** | | | |
| OCT3 WT | 130 ± 32 | 125 ± 9 | 113 ± 16 |
| **Clearance Rate** | | | |
| OCT3 WT | 78 ± 16 | 98 ± 18 | 57 ± 8 |
| **Red:Ox Ratio** | | | |
| OCT3 WT | 115 ± 33 | 86 ± 7 | 89 ± 4 |

Peak amplitude of amphetamine-evoked dopamine release was attenuated by cocaine in nucleus accumbens core (see **Figure 4** in main article and Supplementary Table S6. Data are mean and SEM *percent change* from baseline amphetamine-evoked dopamine release signal (see Supplementary Table S6 for absolute values). * p < 0.05, two-tailed Mann Whitney test compared to aCSF control group. n = 10-11 from 8-11 males and 0-2 females.

**Supplementary Table S7. Effect of vehicle, D22, and cocaine 15 minutes post-infusion on amphetamine-evoked dopamine release and clearance signal parameters in nucleus accumbens core of OCT3 wildtype mice (absolute change from baseline).**

|  | **aCSF** (n = 11) | | **D22** (n = 10) | | **Cocaine** (n = 11) | |
| --- | --- | --- | --- | --- | --- | --- |
|  | **Pre-** | **Post-** | **Pre-** | **Post-** | **Pre-** | **Post-** |
| **Peak Dopamine Release (µM)** | | | | | | |
| OCT3 WT | 0.38 ± 0.04 | 0.34 ± 0.05 | 0.41 ± 0.06 | 0.41 ± 0.06 | 0.46 ± 0.10 | 0.29 ± 0.06 ** |
| **Rise Time (s)** | | | | | | |
| OCT3 WT | 316 ± 36 | 543 ± 228 | 280 ± 35 | 363 ± 79 | 230 ± 37 | 283 ± 49 |
| **Clearance Time, t_80_ (s)** | | | | | | |
| OCT3 WT | 1031 ± 87 | 1168 ± 178 | 813 ± 139 | 1010 ± 165 * | 959 ± 101 | 994 ± 119 |
| **Clearance Rate (nM/s)** | | | | | | |
| OCT3 WT | 0.4 ± 0.1 | 0.3 ± 0.1 | 0.6 ± 0.1 | 0.6 ± 0.2 | 0.4 ± 0.1 | 0.2 ± 0.1 ** |
| **Red:Ox Ratio** | | | | | | |
| OCT3 WT | 0.45 ± 0.04 | 0.47 ± 0.03 | 0.45 ± 0.06 | 0.40 ± 0.06 | 0.48 ± 0.03 | 0.43 ± 0.03 * |

The signal parameter most sensitive to the effects of cocaine in nucleus accumbens was peak dopamine release. Cocaine significantly decreased peak dopamine release (~0.17 µM decrease) in wildtype mice. Data are mean and SEM. * p < 0.05, ** p < 0.01, two-tailed paired t-test or Mann Whitney test (depending on normality) comparing pre- versus post- signal parameter. n = 10-11 from 8-11 males and 0-2 females.

**SUPPLEMENTARY REFERENCES**

1. Zwart R, Verhaagh S, Buitelaar M, Popp-Snijders C, Barlow DP. Impaired activity of the extraneuronal monoamine transporter system known as uptake-2 in Orct3/Slc22a3-deficient mice. Mol Cell Biol. 2001;21(13):4188–96.

2. Ventura A, Kirsch D, McLaughlin M, Tuveson D, Grimm J, Lintault L, et al. Restoration of p53 function leads to tumor regression in vivo. Nature. 2007;445(7128):661–5.

3. Mayer FP, Schmid D, Owens WA, Gould GG, Apuschkin M, Kudlacek O, et al. An unsuspected role for organic cation transporter 3 in the actions of amphetamine. Neuropsychopharmacol. 2018;43:2408–17.

4. Clauss N, Mayer F, Owens W, Vitela M, Clarke K, Bowman M, et al. Ethanol inhibits dopamine uptake via organic cation transporter 3: Implications for ethanol and cocaine co-abuse. Mol Psychiatry. 2023;28:2934–45.

5. Seaman Jr RW, Collins GT. Impact of morphine dependence and withdrawal on the reinforcing effectiveness of fentanyl, cocaine, and methamphetamine in rats. Front Pharmacol. 2021;12:691700.

6. Gannon BM, Sulima A, Rice KC, Collins GT. Inhibition of cocaine and 3,4-methylenedioxypyrovalerone (MDPV) self-administration by lorcaserin is mediated by 5-HT2C receptors in rats. J Pharmacol Exp Ther. 2018;364(2):359–66.
